# Association between short-term systemic use of glucocorticoids and prognosis of cardiogenic shock: a retrospective analysis

**DOI:** 10.1186/s12871-023-02131-y

**Published:** 2023-05-18

**Authors:** Hua-Ping Fan, Yan Zhou, Yu Zhou, Jun Jin, Tian-Yang Hu

**Affiliations:** 1 Department of Cardiology, 63650 Military Hospital, Urumqi, Xinjiang 841700 China; 2 Department of Ophthalmology, The First People’s Hospital of Ziyang, Sichuan, 641300 China; 3 Institute of Cardiovascular Diseases, Xinqiao Hospital, Army Medical University, Chongqing, 400038 China; 4 Precision Medicine Center, The Second Affiliated Hospital of Chongqing Medical University, 74 Linjiang Road, Yuzhong District, Chongqing, 400010 China

**Keywords:** Cardiogenic shock, Glucocorticoids, Adverse effects, Epidemiology, MIMIC-IV database

## Abstract

**Objective:**

To investigate the prescription rate of short-term systemic use of glucocorticoids during hospitalization in patients with cardiogenic shock (CS), and outcomes related with glucocorticoid use.

**Methods:**

We extracted patients' information from the Medical Information Mart for Intensive Care IV version 2.0 (MIMIC-IV v2.0) database. The primary endpoint was 90-day all-cause mortality. Secondary safety endpoints were infection identified by bacterial culture and at least one episode of hyperglycemia after ICU admission. Propensity score matching (PSM) was used to balance baseline characteristics. The difference in cumulative mortality rate between these treated with and without glucocorticoids was assessed by Kaplan–Meier curve with log-rank test. Independent risk factors for endpoints were identified by Cox or Logistic regression analysis.

**Results:**

A total of 1528 patients were enrolled, and one-sixth of these patients received short-term systemic therapy of glucocorticoids during hospitalization. These conditions, including rapid heart rate, the presence of rheumatic disease, chronic pulmonary disease and septic shock, high lactate level, the requirements of mechanical ventilation and continuous renal replacement therapy, were associated with an increase in glucocorticoid administration (all *P* ≤ 0.024). During a follow-up of 90 days, the cumulative mortality rate in patients treated with glucocorticoids was significantly higher than that in these untreated with glucocorticoids (log-rank test, *P* < 0.001). Multivariable Cox regression analysis showed that glucocorticoid use (hazard ratio 1.48, 95% confidence interval [CI] 1.22–1.81; *P* < 0.001) was independently associated with an increased risk for 90-day all-cause mortality. This result was consistent irrespective of age, gender, the presence of myocardial infarction, acute decompensated heart failure and septic shock, and inotrope therapy, but was more evident in low-risk patients as assessed by ICU scoring systems. Additionally, multivariable Logistic regression analysis showed that glucocorticoid exposure was an independent predictor of hyperglycemia (odds ratio 2.14, 95% CI 1.48–3.10; *P* < 0.001), but not infection (odds ratio 1.23, 95% CI 0.88–1.73; *P* = 0.221). After PSM, glucocorticoid therapy was also significantly related with increased risks of 90-day mortality and hyperglycemia.

**Conclusions:**

Real-world data showed that short-term systemic use of glucocorticoids was common in CS patients. Importantly, these prescriptions were associated with increased risks of adverse events.

**Supplementary Information:**

The online version contains supplementary material available at 10.1186/s12871-023-02131-y.

## Introduction

Cardiogenic shock (CS) is the advanced stage of heart failure and is a clinical syndrome characterized by pump failure and low cardiac output, resulting in hypoxic tissue injury and multiple organ dysfunction [1–4]. What leads to CS is attributed to numerous causes, and the most common are myocardial infarction (MI) and acute compensated heart failure (ADHF) [1, 2, 4]. Despite the considerable progress in revascularization and mechanical circulation support therapy, the overall prognosis of CS remains poor, and more than one-third of these patients died during hospitalization [5]. Although the substantive characteristics of CS are hemodynamic disorders, its prognosis depends on metabolic dysfunction and inflammatory activation caused by tissue hypoperfusion [6].

Previous studies showed that CS is associated with a reduced response of the hypothalamic–pituitary–adrenal axis that works together to regulate cortisol production [7–9]. Glucocorticoids have a potent anti-inflammatory effect and have been used to treat a variety of cardiovascular diseases [10]. Moreover, glucocorticoids can maintain vascular tone, enhance cardiac contractility, and reduce the dose titration of vasopressors, and they are essential for the regulation of blood pressure and blood flow [11–13]. These mechanisms seem to indicate that glucocorticoid therapy might be beneficial to CS patients. However, glucocorticoids can act as a double-edged sword, and their therapeutic advantages are at the cost of consequent side effects, such as sepsis, thrombosis, and metabolic disorders, even in a short-term duration of treatment [14, 15]. In addition, glucocorticoids are assumed to cause sodium and water retention and worsen heart failure because the molecular structure of glucocorticoids is similar to that of mineralocorticoids, while a small number of studies showed that glucocorticoids might enhance diuretic effects and improve renal function in ADHF patients [16, 17]. Indeed, clinical evidence about the therapeutic effectiveness of glucocorticoids on CS patients remains controversial. Some case reports demonstrated that glucocorticoids might help CS patients recover [18–20], whereas a small study including 35 patients showed that glucocorticoid use was associated with an increased risk of mortality [9].

Given these dilemmas, even though international guideline has recommended that systemic glucocorticoid use should be administrated with caution in heart failure [21], a significant proportion of these patients were prescribed steroids [22]. Therefore, it is crucial to elucidate the effects of glucocorticoid use in terms of survival for CS patients. For this purpose, we used the Medical Information Mart for Intensive Care IV version 2.0 (MIMIC-IV v2.0) database, which is publicly available regarding critical illness patients, firstly to explore the motivation of physicians to choose short-term systemic use of glucocorticoids in CS, and next to compare side effects and 90-day all-cause mortality who received these medications versus those who did not.

## Methods

### Data source

This study was conducted in accordance with the guideline of the Strengthening the Reporting of Observational Studies in Epidemiology (STROBE) Statement [23]. We retrospectively obtained observational data based on the MIMIC-IV v2.0 database that contains more than 300,000 patients admitted to the critical care units of the Beth Israel Deaconess Medical Center in a tertiary university hospital located in Boston, Massachusetts, USA between 2008 and 2019. This database includes patients' information about demographic characteristics, vital signs, laboratory results, diagnoses, nursing labels, medication prescriptions, liquid balance, and procedure events. Additionally, follow-up records for all-cause mortality were available within one year.

### Study population

We included patients with the diagnoses of CS using the International Classification of Diseases (ICD) of 9^th^ and 10^th^ (Additional file 1: Table S1) revision codes. Exclusion criteria included: (1) multiple admissions and multiple intensive care unit (ICU) stays; (2) patients who aged < 18 years; (3) < 2 days of hospital stay; (4) glucocorticoid exposure beyond 30 days after admission; (5) a history of steroid use except the inhaled (Additional file 1: Table S1).

### Data extraction

The in-hospital information for the included patients was extracted through Structured Query Language. Short-term systemic glucocorticoid exposure was defined as using oral or intravenous glucocorticoid therapy after admission within 30 days, including hydrocortisone, methylprednisolone, prednisone, and dexamethasone. For comparison purposes, a prednisone equivalent dose for each type of glucocorticoid was calculated according to a formula [24]. The closest data to the point of ICU admission were recorded, including vital signs and laboratory results. Comorbidities were searched using ICD codes. We also extracted information about demographic characteristics, common medications, procedure events, and follow-up data. Demographic characteristics included age, gender, weight, and height. Vital signs included heart rate and systolic/diastolic blood pressure. Comorbidities included hypertension, diabetes, dyslipidemia, chronic kidney disease, rheumatic disease, chronic pulmonary disease, MI, cardiac arrest, septic shock, and ADHF. Laboratory results included arterial lactate, white blood cell count, red blood cell count, blood platelet count, hemoglobin, serum alanine aminotransferase, serum aspartate aminotransferase, serum albumin, serum creatinine, blood urea nitrogen, bicarbonate, arterial blood gas value, serum glucose, serum electrolytes, and coagulation function. Medications included aspirin, clopidogrel, ticagrelor, prasugrel, statins, dopamine, dobutamine, norepinephrine, phenylephrine, and milrinone. Procedure events included mechanical ventilation, continuous renal replacement therapy (CRRT), percutaneous coronary intervention, coronary artery bypass grafting, and mechanical circulatory support (MCS), such as intra-aortic balloon pump, extracorporeal membrane oxygenation, and Impella device. Additionally, the first-time scores for disease severity, including the Charlson comorbidity index, sequential organ failure assessment (SOFA) score, oxford acute severity of illness score (OASIS), acute physiology score III (APS III), and logistic organ dysfunction system (LODS), were recorded.

### Endpoints definition

The primary endpoint was 90-day all-cause mortality. Secondary safety endpoints included infection and hyperglycemia. Infection was identified by bacterial culture, and hyperglycemia was defined as at least one episode of random serum glucose ≥ 207 mg/dL after ICU admission [25].

### Statistical analysis

Continuous variables were reported as mean ± standard deviation (SD) if they were normally distributed, and as median with interquartile range (IQR) otherwise. Statistical difference was assessed by student's t-test or Wilcoxon rank-sum test. Categorical variables were reported as number and percentage, and were assessed by Pearson's chi-square test. The missing value for continuous variables was estimated by random forest [26], and was imputed 5 times (the mice package in R software). Propensity score matching (PSM) using a 1-to-2 nearest neighbor method with a caliper of 0.05 was applied to balance baseline characteristics. Calculation of propensity scores were on account of these variables: age, male, body mass index, heart rate, Charlson comorbidity index, OASIS, APS III, diabetes, dyslipidemia, chronic pulmonary disease, rheumatic disease, ADHF, septic shock, lactate, white blood cell, serum albumin, alanine aminotransferase, blood urea nitrogen, serum glucose, serum sodium, serum potassium, serum calcium, international normalized ratio, inotropes, CRRT, revascularization, MCS, and mechanical ventilation. The difference of baseline characteristics between groups after PSM were assessed by standardized mean differences (SMDs), and < 0.1 of SMD [27] suggested that their baseline characteristics were well balanced. Kaplan–Meier curve provided a visual image to compare the difference in cumulative mortality rate, that was quantitatively evaluated by log-rank test. Multicollinearity in the multivariable linear regression analyses was assessed by variance inflation factor (VIF). VIF > 5 suggested the possibility of multicollinearity [28]. The multicollinearity was trivial when base excess and hemoglobin were removed from the regression model (Additional file 1: Table S2). Multivariable Cox or Logistics regression analyses were used to identity independent risk factors, and effect sizes were summarized as hazard ratios (HRs) or odds ratios (ORs) with 95% confidence intervals (CIs). We selected variables with a P-value of < 0.05 in univariable regression analyses for inclusion in a multivariable regression model. Subgroup analyses and interactions were performed based on age (≥ 75 years vs. < 75 years), gender (male vs. female), MI (yes vs. no), ADHF (yes vs. no), septic shock (yes vs. no), inotropes (yes vs. no), Charlson comorbidity index (≥ 7 vs. < 7), SOFA score (≥ 2 vs. < 2), APS III (≥ 59 vs. < 59), OASIS (≥ 37 vs. < 37), and LODS (≥ 7 vs. < 7). Additionally, three sets of sensitivity analyses were conducted to verify the robustness of our results. Firstly, we analyzed whether the effects of glucocorticoids on mortality varied with different daily dosage, cumulative dosages and treatment duration. Secondly, considering that the primary purpose of glucocorticoid administration for some clinical physicians was to treat comorbidities rather than the disease itself, sensitivity analysis was conducted by excluding CS patients with chronic pulmonary disease and rheumatic disease. Thirdly, since all findings in the study were based on the data after multiple imputations, raw data was further analyzed. All statistical analyses were conducted in Stata version 15.1 (StataCorp LLC, College Station, TX77845, USA) and R 4.2.1 (The R Foundation for Statistical Computing) software. Statistical significance was set at a *P*-value of < 0.05.

## Results

### Baseline characteristics

**Fig. 1:**
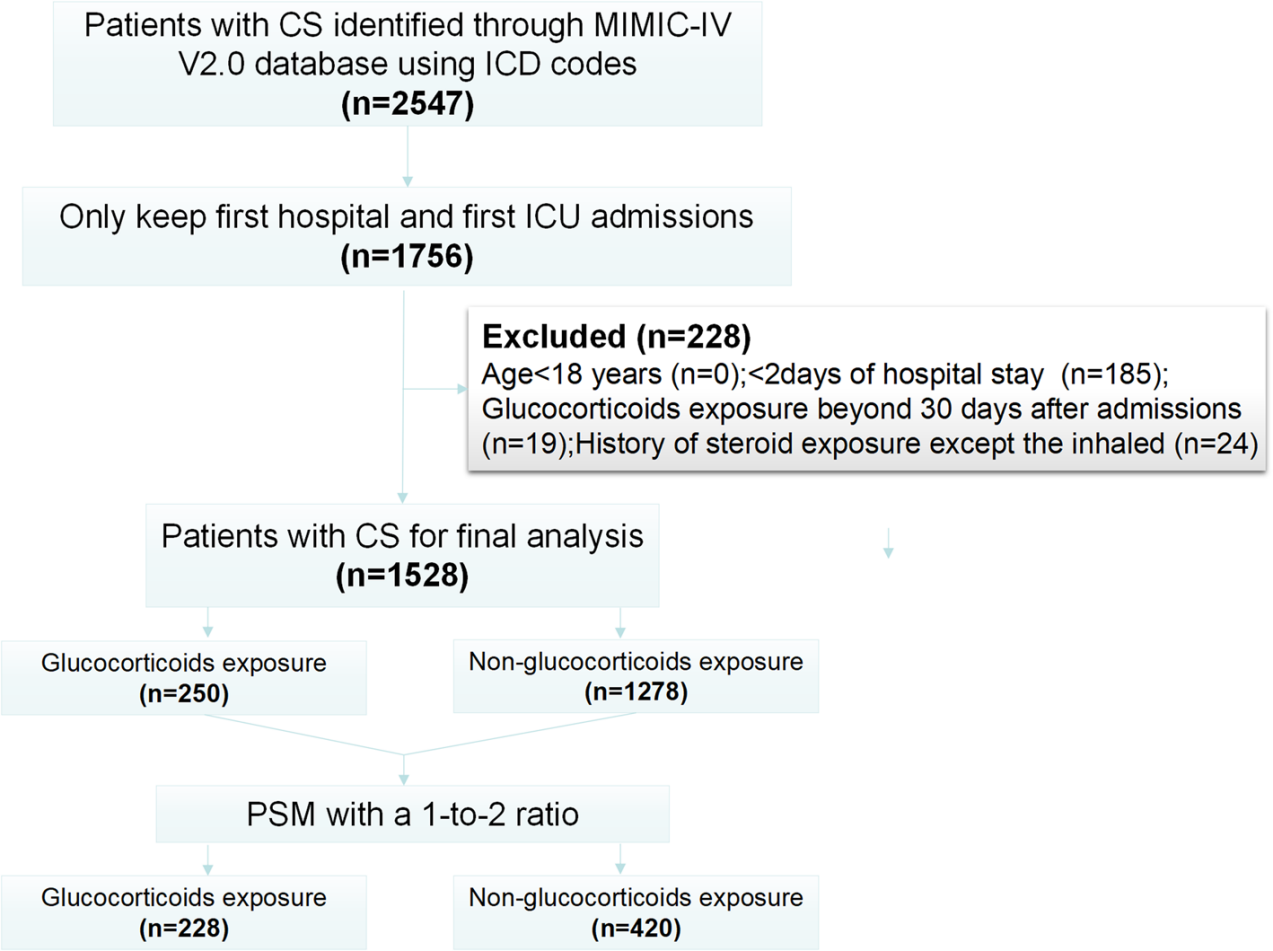
Flowchart of patient selection. CS, cardiogenic shock; MIMIC-IV, Medical Information Mart for Intensive Care IV; ICD, International Classification of Diseases; ICU, intensive care unit; PSM, propensity score matching

We screened 2547 records with the diagnoses of CS in the database and excluded 1019 records (Fig. 1). Finally, 1528 patients were enrolled in the analyses. Table 1 shows the baseline characteristics before and after PSM. In the original cohort, the median age was 72.0 years and males accounted for 60.2%. The underlying causes of CS were attributed to MI in 49.9% and ADHF in 59.1%, and 22.1% had none of these causes. Mechanical ventilation and vasopressors were required for most of these patients. A total of 250 patients received glucocorticoid therapy, accounting for 16.4%. In glucocorticoid users, the median exposure duration was 4 days (IQR, 2–7 days), with a median daily dosage of 50 mg (IQR, 30–80 mg/day) and a median cumulative dosage of 180 mg (IQR, 75–400 mg). Baseline characteristics between those treated and untreated with glucocorticoids were unbalanced in several variables, including age, gender, heart rate, all ICU scoring systems except SOFA score, comorbidities (i.e., dyslipidemia, rheumatic disease, chronic pulmonary disease, cardiac arrest, septic shock, and ADHF), lactate, blood gas (i.e., pH and base excess), admission serum glucose level, all medications, and all procedure events except MCS. Multivariable Logistic regression analysis showed that advanced age and inotrope use were associated with reduced use of glucocorticoids (Table 2; all *P* ≤ 0.027), and there was an increase in glucocorticoid administration when patients had rapid heart rate, high lactate level, the presence of rheumatic disease, chronic pulmonary disease and septic shock, and therapy with CRRT and mechanical ventilation (all *P* ≤ 0.024). With regard to endpoints, glucocorticoid users had significantly higher event rates for 90-day all-cause mortality (58.0% vs. 35.6%; *P* < 0.001), infection (30.4% vs. 19.1%; *P* < 0.001) and hyperglycemia (69.2% vs. 49.1%; *P* < 0.001) than non-glucocorticoid users.

**Table 1:** Baseline characteristics of patients with cardiogenic shock

	**Before PSM**				**After PSM**		
	**Non-glucocorticoid (*n* = 1278)**	**Glucocorticoid (*n* = 250)**	**Missing values**	***P* value**	**Non-glucocorticoid (*n* = 420)**	**Glucocorticoid (*n* = 228)**	***P* value**
**Age (years), median (IQR)**	72.6 (62.1, 81.2)	69.3 (60.1, 79.0)	0 (0)	0.015	72.4 (61.6, 79.7)	69.7 (61.0, 79.6)	0.168
**Male, n (%)**	790 (61.8)	130 (52.0)		0.005	226 (53.8)	121 (53.1)	0.922
**BMI (Kg/m**^**2**^**), median (IQR)**	27.7 (24.3, 32.1)	27.2 (23.8, 31.7)	176 (11.5)	0.399	27.4 (23.9, 31.6)	27.1 (23.8, 31.4)	0.783
**ICU scoring systems**							
Charlson comorbidity index, mean ± SD	6.9 ± 2.6	7.3 ± 2.8	0 (0)	0.015	7.3 ± 2.8	7.3 ± 2.7	0.985
SOFA, median (IQR)	3 (1, 5)	3 (1, 5)	0 (0)	0.513	3 (1, 5)	3 (1, 4)	0.716
OASIS, mean ± SD	36.8 ± 10.0	39.4 ± 10.0	0 (0)	< 0.001	39.1 ± 9.8	38.9 ± 10.0	0.831
APS III, mean ± SD	62.3 ± 27.2	72.1 ± 27.4	0 (0)	< 0.001	69.9 ± 30.5	70.2 ± 26.9	0.798
LODS, median (IQR)	7 (4, 10)	8 (6, 11)	0 (0)	< 0.001	8 (5, 11)	8 (6, 11)	0.899
**Vital signs**							
SBP (mmHg), mean ± SD	119.2 ± 22.0	111.5 ± 21.4	4 (0.3)	0.773	112.1 ± 22.5	110.8 ± 20.4	0.475
DBP (mmHg), mean ± SD	65.9 ± 18.7	66.2 ± 18.6	4 (0.3)	0.830	65.0 ± 18.6	66.3 ± 18.9	0.374
Heart rate (bpm), mean ± SD	90.6 ± 20.8	96.2 ± 23.1	0 (0)	< 0.001	93.8 ± 22.2	95.5 ± 22.8	0.344
**Comorbidities**							
Hypertension, n (%)	372 (29.1)	69 (27.6)		0.686	109 (26.0)	67 (29.4)	0.398
Diabetes, n (%)	477 (37.3)	85 (34.0)		0.355	141 (33.6)	75 (32.9)	0.930
Dyslipidemia, n (%)	660 (51.6)	108 (43.2)		0.018	192 (45.7)	101 (44.3)	0.792
CKD, n (%)	442 (34.6)	91 (36.4)		0.633	149 (35.5)	79 (34.6)	0.901
Rheumatic disease, n (%)	28 (2.2)	19 (7.2)		< 0.001	19 (4.5)	11 (4.8)	> 0.999
Chronic pulmonary disease, n (%)	353 (27.6)	99 (39.6)		< 0.001	152 (36.2)	87 (38.2)	0.681
Cardiac arrest, n (%)	150 (11.7)	43 (17.2)		0.023	69 (16.4)	40 (71.5)	0.801
Septic shock, n (%)	193 (15.1)	78 (31.2)		< 0.001	107 (25.5)	63 (27.6)	0.616
MI, n (%)	642 (50.2)	121 (48.4)		0.644	214 (51.0)	113 (49.6)	0.798
ADHF, n (%)	774 (60.6)	129 (51.6)		0.010	221 (52.6)	121 (53.1)	0.978
**Laboratory tests**							
Lactate (mmol/L), median (IQR)	1.9 (1.3, 3.1)	2.4 (1.5, 4.1)	58 (3.8)	< 0.001	2.2 (1.5, 3.9)	2.4 (1.5, 4.0)	0.519
WBC (× 10^9^/L), median (IQR)	11.6 (8.1, 16.0)	11.5 (7.5, 16.2)	2 (0.1)	0.373	11.7 (8.1, 16.7)	11.7 (7.8, 16.5)	0.602
RBC (× 10^12^/L), mean ± SD	3.9 ± 0.8	3.8 ± 0.8	2 (0.1)	0.127	3.8 ± 0.8	3.8 ± 0.8	0.570
Hemoglobin (g/L), mean ± SD	11.6 ± 2.4	11.4 ± 2.3	2 (0.1)	0.168	11.3 ± 2.3	11.4 ± 2.3	0.576
Platelet (× 10^9^/L), median (IQR)	202 (154, 262)	189 (146, 257)	2 (0.1)	0.044	201 (154, 269)	192 (147, 261)	0.113
ALT (IU/L), mean ± SD	39 (20, 118)	47 (22, 127)	97 (6.3)	0.148	43 (21, 124)	47 (23, 126)	0.652
AST (IU/L), mean ± SD	62 (29, 221)	74 (31, 181)	102 (6.3)	0.851	76 (32, 238)	80 (33, 182)	0.513
Albumin (g/dL), mean ± SD	3.2 ± 0.6	3.0 ± 0.6	325 (20.8)	< 0.001	3.1 ± 0.6	3.0 ± 0.6	0.611
Creatinine (mg/dL), median (IQR)	1.4 (1.0, 2.0)	1.3 (1.0, 2.0)	1 (0.1)	0.945	1.4 (0.9, 2.0)	1.3 (1.0, 2.0)	0.975
BUN (mg/dL), median (IQR)	28 (19, 46)	28 (19, 42)	1 (0.1)	0.535	27 (18, 46)	28 (19, 41)	0.968
Bicarbonate (mmol/L), mean ± SD	22.0 ± 4.7	21.8 ± 5.4	1 (0.1)	0.527	21.6 ± 4.8	21.9 ± 5.4	0.487
pH, mean ± SD	7.34 ± 0.1	7.31 ± 0.1	84 (5.5)	0.021	7.31 ± 0.2	7.31 ± 0.1	0.937
BE (mmol/L), median (IQR)	-2 (-6, 0)	-4 (-8, 0)	84 (5.5)	< 0.001	-3 (-7, 0)	-4 (-8, 0)	0.360
Glucose (mg/dL), median (IQR)	143 (112, 196)	156 (116, 216)	1 (0.1)	0.044	149 (115, 213)	157 (118, 218)	0.732
Calcium (mg/dL), mean ± SD	8.5 ± 0.9	8.3 ± 1.0	2 (0.1)	0.003	8.3 ± 1.0	8.3 ± 1.0	0.930
Magnesium (mg/dL), mean ± SD	2.1 ± 0.4	2.1 ± 0.6	1 (0.1)	0.204	2.1 ± 0.5	2.1 ± 0.6	0.499
Chloride (mmol/L), mean ± SD	101.9 ± 6.7	102.1 ± 7.2	1 (0.1)	0.535	102.4 ± 6.5	102.2 ± 7.4	0.634
Sodium (mmol/L), mean ± SD	137.6 ± 5.1	137.9 ± 5.6	1 (0.1)	0.320	137.9 ± 5.0	137.8 ± 5.7	0.866
Potassium (mmol/L), mean ± SD	4.5 ± 0.9	4.5 ± 0.9	1 (0.1)	0.355	4.5 ± 0.9	4.5 ± 0.9	0.851
INR, median (IQR)	1.3 (1.2, 1.8)	1.3 (1.2, 1.6)	9 (5.9)	0.744	1.3 (1.2, 1.7)	1.3 (1.2, 1.6)	0.571
PTT (s), median (IQR)	35.4 (29.1, 55.0)	34.7 (28.3, 50.4)	8 (5.2)	0.163	34.5 (28.7, 52.3)	35.2 (28.6, 53.3)	0.989
**Medications**							
Antiplatelet, n (%)	1044 (81.7)	186 (74.4)		0.010	335 (79.8)	173 (75.9)	0.295
Statins, n (%)	919 (71.9)	160 (64.0)		0.015	279 (66.4)	148 (64.9)	0.763
Vasopressors, n (%)	949 (74.3)	211 (84.4)		0.001	346 (82.4)	190 (83.3)	0.844
Inotropes, n (%)	487 (38.1)	77 (30.8)		0.034	139 (33.1)	75 (32.9)	> 0.999
**Procedure events**							
MCS, n (%)	93 (7.3)	27 (10.8)		0.078	55 (13.1)	25 (11.0)	0.508
CRRT, n (%)	151 (11.8)	67 (26.8)		< 0.001	83 (19.8)	52 (22.8)	0.418
Mechanical ventilation, n (%)	763 (59.7)	186 (74.4)		< 0.001	319 (76.0)	166 (72.8)	0.432
Revascularization, n (%)	413 (32.3)	56 (22.4)		0.002	106 (25.2)	55 (24.1)	0.827
**90-day all-cause mortality, n (%)**	455 (35.6)	145 (58.0)		< 0.001	191 (45.5)	130 (57.0)	0.005
**Infection, n (%)**	244 (19.1)	76 (30.4)		< 0.001	106 (25.2)	68 (29.8)	0.208
**Hyperglycemia, n (%)**	628 (49.1)	173 (69.2)		< 0.001	226 (53.8)	156 (68.4)	< 0.001

*PSM* Propensity score matching, *BMI* Body mass index, *ICU* Intensive care unit, *SOFA* Sequential organ failure assessment, *OASIS* Oxford acute severity of illness score, *APS III* Acute physiology score III, *LODS* Logistic organ dysfunction system, *SBP* Systolic blood pressure, *DBP* Diastolic blood pressure, *CKD* Chronic kidney disease, *MI* Myocardial infarction, *ADHF* Acute decompensated heart failure, *WBC* White blood cell, *RBC* Red blood cell, *ALT* Alanine aminotransferase, *AST* Aspartate aminotransferase, *BUN* Blood urea nitrogen, *BE* Base excess, *INR* International normalized ratio, *PTT* Partial thromboplastin time, *MCS* Mechanical circulatory support, *CRRT* Continuous renal replacement therapy

**Table 2:** Logistics regression analysis of factors associated with glucocorticoid use

	**Univariable analysis**		**Multivariable analysis**	
	**OR (95% CI)**	***P* value**	**OR (95% CI)**	***P* value**
**Age, per 1 year**	0.99 (0.98–1.00)	0.033	0.99 (0.97–1.00)	0.027
**Male**	0.67 (0.51–0.88)	0.004	0.70 (0.51–0.96)	0.024
BMI, per 1 kg/m^2^	0.99 (0.98–1.02)	0.615	–	
**Charlson comorbidity index, per 1 score**	1.07 (1.01–1.12)	0.015	1.11 (1.04–1.19)	0.003
SOFA, per 1 score	1.03 (0.99–1.09)	0.165	–	
**OASIS, per 1 score**	1.03 (1.01–1.04)	< 0.001	0.97 (0.95–1.00)	0.024
APS III, per 1 score	1.01 (1.01–1.02)	< 0.001	1.00 (0.99–1.01)	0.420
LODS, per 1 score	1.09 (1.05–1.13)	< 0.001	1.00 (0.92–1.08)	0.959
SBP, per 1 mmHg	1.00 (0.99–1.01)	0.773	–	
DBP, per 1 mmHg	1.00 (0.99–1.01)	0.830	–	
**Heart rate, per 1 bpm**	1.01 (1.01–1.02)	< 0.001	1.01 (1.00–1.02)	0.001
Hypertension	0.93 (0.69–1.26)	0.630	–	
Diabetes	0.87 (0.65–1.15)	0.319	–	
Dyslipidemia	0.71 (0.54–0.94)	0.015	0.85 (0.62–1.16)	0.297
CKD	1.08 (0.82–1.44)	0.582	–	
**Rheumatic disease**	3.46 (1.88–6.36)	< 0.001	3.61 (1.84–7.10)	< 0.001
**Chronic pulmonary disease**	1.72 (1.30–2.28)	< 0.001	1.61 (1.17–2.21)	0.004
ADHF	0.69 (0.53–0.91)	0.009	0.80 (0.58–1.09)	0.152
MI	0.93 (0.71–1.22)	0.596	–	
Cardiac arrest	1.56 (1.08–2.26)	0.018	1.27 (0.83–1.94)	0.271
**Septic shock**	2.55 (1.87–3.47)	< 0.001	1.70 (1.19–2.44)	0.004
**Lactate, per 1 mmol/L**	1.12 (1.07–1.18)	< 0.001	1.07 (1.01–1.14)	0.024
WBC, per 1 × 10^9^/L	0.99 (0.97–1.01)	0.388	–	
Platelet, per 1 × 10^9^/L	1.00 (1.00–1.00)	0.199	–	
RBC, per 1 × 10^12^/L	0.88 (0.74–1.04)	0.127	–	
Hemoglobin, per 1 g/L	0.96 (0.91–1.02)	0.168	–	
AST, per 1 IU/L	1.00 (1.00–1.00)	0.260	–	
ALT, per 1 IU/L	1.00 (1.00–1.00)	0.881	–	
Albumin, per 1 g/dL	0.62 (0.49–0.78)	< 0.001	0.86 (0.66–1.11)	0.234
BUN, per 1 mg/dL	1.00 (0.99–1.00)	0.312	–	
Creatinine, per 1 mg/dL	1.03 (0.95–1.13)	0.456	–	
Bicarbonate, per 1 mmol/L	0.99 (0.96–1.02)	0.527	–	
pH, per 0.01	0.55 (0.24–1.26)	0.155	–	
BE, per 1 mmol/L	0.99 (0.99–1.00)	0.267	–	
Glucose, per 1 mg/dL	1.00 (1.00–1.00)	0.020	1.00 (1.00–1.00)	0.278
Sodium, per 1 mmol/L	1.01 (0.99–1.04)	0.320	–	
Potassium, per 1 mmol/L	1.07 (0.92–1.25)	0.355	–	
Chloride, per 1 mmol/L	1.01 (0.99–1.03)	0.534	–	
Calcium, per 1 mg/dL	0.80 (0.69–0.93)	0.003	0.94 (0.79–1.10)	0.431
Magnesium, per 1 mg/dL	0.81 (0.59–1.12)	0.202	–	
INR, per 0.1	0.91 (0.80–1.04)	0.172	–	
PTT, per 1 s	1.00 (0.99–1.00)	0.390	–	
Antiplatelet	0.65 (0.47–0.89)	0.008	0.89 (0.59–1.34)	0.579
Statins	0.69 (0.52–0.92)	0.012	0.99 (0.67–1.44)	0.938
**Inotropes**	0.72 (0.54–0.97)	0.029	0.57 (0.41–0.80)	0.001
Vasopressors	1.88 (1.30–2.70)	0.001	1.15 (0.73–1.81)	0.538
MCS	1.54 (0.98–2.42)	0.060	–	
**CRRT**	2.73 (1.97–3.79)	< 0.001	2.25 (1.51–3.34)	< 0.001
Revascularization	0.60 (0.44–0.83)	0.002	0.69 (0.48–1.01)	0.057
**Mechanical ventilation**	1.96 (1.45–2.66)	< 0.001	1.95 (1.25–3.04)	0.003

*BMI* Body mass index, *SOFA* Sequential organ failure assessment, *OASIS* Oxford acute severity of illness score, *APS III* Acute physiology score III, *LODS* Logistic organ dysfunction system, *SBP* Systolic blood pressure, *DBP* Diastolic blood pressure, *CKD* Chronic kidney disease, *ADHF* Acute decompensated heart failure, *MI* Myocardial infarction, *WBC* White blood cell, *RBC* Red blood cell, *AST* Aspartate aminotransferase, *ALT* Alanine aminotransferase, *BUN* Blood urea nitrogen, *BE* Base excess, *INR* International normalized ratio, *PTT* Partial thromboplastin time, *MCS* Mechanical circulatory support, *CRRT* Continuous renal replacement therapy, *OR* Odds ratio, *CI*, Confidence interval

In the post-matched cohort, 228 patients received glucocorticoid therapy and 420 patients did not. The SMDs of all variables were < 0.1 (Additional file 1: Figure S1), suggesting the baseline characteristics of this cohort were well-balanced. The event rates for all-cause mortality (57.0% vs. 45.5%; *P* = 0.005) and hyperglycemia (68.4% vs. 53.8%; *P* < 0.001) were also significantly higher in glucocorticoid users than that in non-glucocorticoid users, but not for infection (29.8% vs. 25.2%; *P* = 0.208).

### Association of glucocorticoid exposure with primary endpoint

**Fig. 2:**
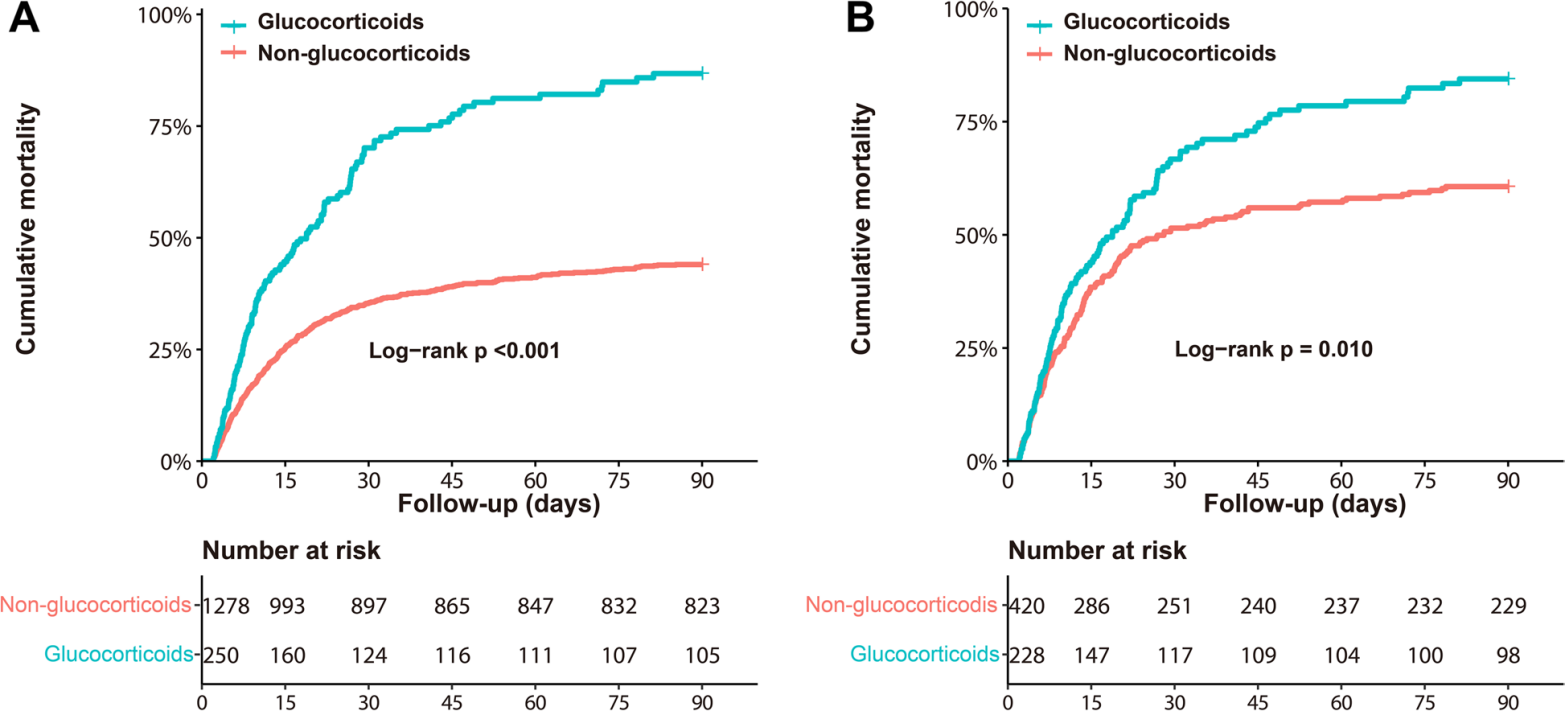
Kaplan–Meier survival curve for 90-day all-cause mortality in cardiogenic shock patients treated with and without glucocorticoids. **A** pre-matched cohort; (**B**) post-matched cohort

The cumulative 90-day all-cause mortality rate of glucocorticoid users was higher than that of non-glucocorticoid users in the pre-matched cohort (Fig. 2A; log-rank test, *P* < 0.001), which was in accordance with the result in the post-matched cohort (Fig. 2B; log-rank test, *P* = 0.010). In the pre-matched cohort, multivariable Cox regression analysis showed that glucocorticoid use (HR 1.48, 95% CI 1.22–1.81; *P* < 0.001) was significantly associated with an increased risk for 90-day mortality (Table 3). In the post-matched cohort, a similar ratio (HR 1.42, 95% CI 1.13–1.79; *P* = 0.003) was observed after adjustment by multivariable Cox regression analysis (Table 4).

**Table 3:** Cox regression analysis for 90-day all-cause mortality in cardiogenic shock patients before matching

	Univariable regression		Multivariable regression	
	HR (95% CI)	P value	HR (95% CI)	*P* value
**Age, per 1 year**	1.03 (1.02–1.04)	< 0.001	1.03 (1.02–1.04)	< 0.001
Male	0.87 (0.74–1.02)	0.096	–	
BMI, per 1 kg/m^2^	0.99 (0.98–1.00)	0.124	–	
**Charlson comorbidity index, per 1 score**	1.18 (1.14–1.21)	< 0.001	1.09 (1.05–1.14)	< 0.001
SOFA, per 1 score	1.04 (1.01–1.07)	0.005	0.98 (0.95–1.01)	0.202
OASIS, per 1 score	1.05 (1.04–1.06)	< 0.001	1.00 (0.98–1.01)	0.554
**APS III, per 1 score**	1.02 (1.02–1.02)	< 0.001	1.01 (1.01–1.02)	< 0.001
LODS, per 1 score	1.15 (1.12–1.17)	< 0.001	1.03 (0.99–1.07)	0.194
SBP, per 1 mmHg	1.00 (0.99–1.00)	0.055	–	
DBP, per 1 mmHg	1.00 (0.99–1.00)	0.052	–	
Heart rate, per 1 bpm	1.01 (1.00–1.01)	0.005	1.00 (1.00–1.01)	0.200
Hypertension	0.79 (0.66–0.95)	0.013	0.87 (0.70–1.09)	0.232
Diabetes	1.12 (0.95–1.32)	0.165	–	
Dyslipidemia	0.98 (0.84–1.15)	0.832	–	
CKD	1.73 (1.47–2.03)	< 0.001	0.98 (0.76–1.25)	0.840
Rheumatic disease	1.07 (0.68–1.69)	0.770	–	
Chronic pulmonary disease	1.14 (0.96–1.36)	0.125	–	
**ADHF**	0.74 (0.63–0.87)	< 0.001	0.68 (0.57–0.81)	< 0.001
**MI**	1.22 (1.04–1.44)	0.014	1.67 (1.38–2.02)	< 0.001
**Cardiac arrest**	1.85 (1.51–2.28)	< 0.001	1.76 (1.41–2.21)	< 0.001
**Septic shock**	2.31 (1.93–2.76)	< 0.001	1.25 (1.01–1.53)	0.036
**Lactate, per 1 mmol/L**	1.13 (1.10–1.16)	< 0.001	1.05 (1.02–1.09)	0.001
WBC, per 1 × 10^9^/L	1.01 (1.00–1.02)	0.131	–	
Platelet, per 1 × 10^9^/L	1.00 (1.00–1.00)	0.151	–	
RBC, per 1 × 10^12^/L	0.78 (0.70–0.86)	< 0.001	0.96 (0.86–1.07)	0.450
Hemoglobin, per 1 g/L	0.91 (0.88–0.94)	< 0.001	–	
AST, per 1 IU/L	1.00 (1.00–1.00)	0.210	–	
ALT, per 1 IU/L	1.00 (1.00–1.00)	0.608	–	
Albumin, per 1 g/dL	0.69 (0.61–0.79)	< 0.001	0.90 (0.77–1.04)	0.154
BUN, per 1 mg/dL	1.01 (1.01–1.01)	< 0.001	1.00 (1.00–1.01)	0.098
Creatinine, per 1 mg/dL	1.12 (1.07–1.17)	< 0.001	0.98 (0.91–1.06)	0.562
Bicarbonate, per 1 mmol/L	0.97 (0.96–0.99)	0.003	1.02 (1.00–1.04)	0.103
pH, per 0.01	0.84 (0.69–1.02)	0.086	–	
BE, per 1 mmol/L	1.00 (0.99–1.00)	0.048	–	
Glucose, per 1 mg/dL	1.00 (1.00–1.00)	0.001	1.00 (1.00–1.00)	0.426
Sodium, per 1 mmol/L	0.99 (0.98–1.01)	0.344	–	
Potassium, per 1 mmol/L	1.11 (1.02–1.22)	0.014	1.00 (0.91–1.11)	0.971
Chloride, per 1 mmol/L	0.98 (0.97–1.00)	0.007	1.00 (0.98–1.01)	0.600
Calcium, per 1 mg/dL	1.01 (0.92–1.10)	0.906	–	
**Magnesium, per 1 mg/dL**	1.22 (1.03–1.44)	0.020	1.25 (1.07–1.46)	0.005
INR, per 0.1	1.04 (0.99–1.09)	0.142	–	
PTT, per 1 s	1.00 (1.00–1.00)	0.213	–	
**Antiplatelet**	0.60 (0.50–0.72)	< 0.001	0.69 (0.55–0.86)	0.001
**Statins**	0.71 (0.60–0.84)	< 0.001	0.78 (0.63–0.96)	0.020
Inotropes	1.12 (0.95–1.32)	0.179	–	
Vasopressors	1.64 (1.33–2.02)	< 0.001	1.09 (0.85–1.40)	0.483
MCS	1.30 (0.98–1.71)	0.070	–	
**CRRT**	2.26 (1.87–2.73)	< 0.001	1.31 (1.04–1.65)	0.022
**Revascularization**	0.53 (0.43–0.64)	< 0.001	0.62 (0.49–0.78)	< 0.001
Mechanical ventilation	1.15 (0.98–1.36)	0.094	–	
**Glucocorticoids**	1.93 (1.60–2.33)	< 0.001	1.48 (1.22–1.81)	< 0.001

*BMI* Body mass index, *SOFA* Sequential organ failure assessment, *OASIS* Oxford acute severity of illness score, *APS III* Acute physiology score III, *LODS* Logistic organ dysfunction system, *SBP* Systolic blood pressure, *DBP* Diastolic blood pressure, *CKD* Chronic kidney disease, *ADHF* Acute decompensated heart failure, *MI* Myocardial infarction, *WBC* White blood cell, *RBC* Red blood cell, *AST* Aspartate aminotransferase, *ALT* Alanine aminotransferase, *BUN* Blood urea nitrogen, *BE* Base excess, *INR* International normalized ratio, *PTT* Partial thromboplastin time, *MCS* Mechanical circulatory support, *CRRT* Continuous renal replacement therapy, *HR* Hazard ratio, *CI* Confidence interval

**Table 4:** Cox regression analysis for 90-day all-cause mortality in cardiogenic shock patients after matching

	Univariable regression		Multivariable regression	
	HR (95% CI)	P value	HR (95% CI)	*P* value
**Age, per 1 year**	1.03 (1.02–1.04)	< 0.001	1.03 (1.02–1.04)	< 0.001
Male	1.16 (0.93–1.45)	0.178	–	
BMI, per 1 kg/m^2^	1.00 (0.98–1.01)	0.736	–	
**Charlson comorbidity index, per 1 score**	1.13 (1.09–1.18)	< 0.001	1.08 (1.03–1.14)	0.003
SOFA, per 1 score	1.03 (0.99–1.07)	0.167	–	
OASIS, per 1 score	1.05 (1.04–1.06)	< 0.001	1.00 (0.98–1.02)	0.679
**APS III, per 1 score**	1.02 (1.01–1.02)	< 0.001	1.01 (1.00–1.02)	0.011
LODS, per 1 score	1.13 (1.09–1.16)	< 0.001	1.02 (0.96–1.09)	0.443
SBP, per 1 mmHg	0.99 (0.99–1.00)	0.023	0.99 (0.99–1.00)	0.055
DBP, per 1 mmHg	0.99 (0.99–1.00)	0.099	–	
**Heart rate, per 1 bpm**	1.01 (1.00–1.01)	0.013	1.01 (1.00–1.01)	0.003
Hypertension	0.77 (0.60–1.00)	0.051	–	
Diabetes	1.08 (0.86–1.36)	0.498	–	
Dyslipidemia	0.94 (0.76–1.18)	0.602	–	
CKD	1.78 (1.43–2.22)	< 0.001	1.30 (0.97–1.74)	0.076
Rheumatic disease	0.74 (0.42–1.32)	0.313	–	
Chronic pulmonary disease	0.91 (0.72–1.14)	0.398	–	
**ADHF**	0.66 (0.53–0.82)	< 0.001	0.60 (0.47–0.76)	< 0.001
MI	1.23 (0.99–1.53)	0.065	–	
**Cardiac arrest**	1.60 (1.23–2.10)	0.001	1.58 (1.17–2.12)	0.003
**Septic shock**	1.92 (1.53–2.42)	< 0.001	1.32 (1.02–1.70)	0.037
**Lactate, per 1 mmol/L**	1.12 (1.08–1.16)	< 0.001	1.08 (1.03–1.13)	0.001
WBC, per 1 × 10^9^/L	1.01 (0.99–1.02)	0.455	–	
Platelet, per 1 × 10^9^/L	1.00 (1.00–1.00)	0.170	–	
RBC, per 1 × 10^12^/L	0.88 (0.77–1.01)	0.071	–	
Hemoglobin, per 1 g/L	0.97 (0.92–1.02)	0.196	–	
AST, per 1 IU/L	1.00 (1.00–1.00)	< 0.001	1.00 (1.00–1.00)	0.128
ALT, per 1 IU/L	1.00 (1.00–1.00)	0.003	1.00 (1.00–1.00)	0.382
Albumin, per 1 g/dL	0.83 (0.69–0.99)	0.041	0.92 (0.76–1.12)	0.420
BUN, per 1 mg/dL	1.01 (1.01–1.01)	< 0.001	1.01 (1.00–1.01)	0.073
Creatinine, per 1 mg/dL	1.11 (1.05–1.18)	< 0.001	0.92 (0.83–1.02)	0.133
Bicarbonate, per 1 mmol/L	0.96 (0.94–0.99)	0.002	0.99 (0.97–1.02)	0.666
pH, per 0.01	0.95 (0.73–1.22)	0.663	–	
BE, per 1 mmol/L	1.00 (0.99–1.00)	0.519	–	
Glucose, per 1 mg/dL	1.00 (1.00–1.00)	0.182	–	
Sodium, per 1 mmol/L	1.00 (0.98–1.03)	0.660	–	
Potassium, per 1 mmol/L	1.04 (0.93–1.18)	0.469	–	
Chloride, per 1 mmol/L	0.99 (0.98–1.01)	0.385	–	
Calcium, per 1 mg/dL	1.02 (0.91–1.14)	0.707	–	
Magnesium, per 1 mg/dL	1.21 (0.99–1.49)	0.061	–	
INR, per 0.1	1.08 (0.99–1.17)	0.083	–	
PTT, per 1 s	1.00 (1.00–1.00)	0.683	–	
Antiplatelet	0.60 (0.47–0.77)	< 0.001	0.77 (0.58–1.01)	0.059
Statins	0.83 (0.66–1.05)	0.117	–	
Inotropes	1.14 (0.91–1.43)	0.267	–	
Vasopressors	1.51 (1.10–2.08)	0.011	0.96 (0.67–1.38)	0.839
MCS	0.96 (0.68–1.36)	0.829	–	
CRRT	1.73 (1.35–2.20)	< 0.001	1.29 (0.97–1.73)	0.083
Revascularization	0.59 (0.44–0.78)	< 0.001	0.81 (0.59–1.1)	0.180
Mechanical ventilation	1.09 (0.85–1.41)	0.503	–	
**Glucocorticoids**	1.34 (1.07–1.67)	0.010	1.42 (1.13–1.79)	0.003

*BMI* Body mass index, *SOFA* Sequential organ failure assessment, *OASIS* Oxford acute severity of illness score, *APS III* Acute physiology score III, *LODS* Logistic organ dysfunction system, *SBP* Systolic blood pressure, *DBP* Diastolic blood pressure, *CKD* Chronic kidney disease, *ADHF* Acute decompensated heart failure, *MI* Myocardial infarction, *WBC* White blood cell, *RBC* Red blood cell, *AST* Aspartate aminotransferase, *ALT* Alanine aminotransferase, *BUN* Blood urea nitrogen, *BE* Base excess, *INR* International normalized ratio, *PTT* Partial thromboplastin time, *MCS* Mechanical circulatory support, *CRRT* Continuous renal replacement therapy, *HR* Hazard ratio, *CI* Confidence interval

### Association of glucocorticoid exposure with secondary safety endpoints

In the pre-matched cohort, adjusted analysis showed that glucocorticoid use was associated with an increased risk of hyperglycemia (OR 2.14, 95% CI 1.48–3.10; *P* < 0.001), which was consistent with the result (OR 2.36, 95% CI 1.54–3.62; *P* < 0.001) in the post-matched cohort (Addition file 1: Table S3 and S4). However, we did not observe that glucocorticoid exposure was an independent predictor for infection (OR 1.23, 95% CI 0.88–1.73; *P* = 0.221) in multivariable regression analysis, although unadjusted analysis showed that glucocorticoid use was associated with an increased risk of infection (Addition file 1: Table S5).

### Subgroup and sensitivity analyses

**Fig. 3:**
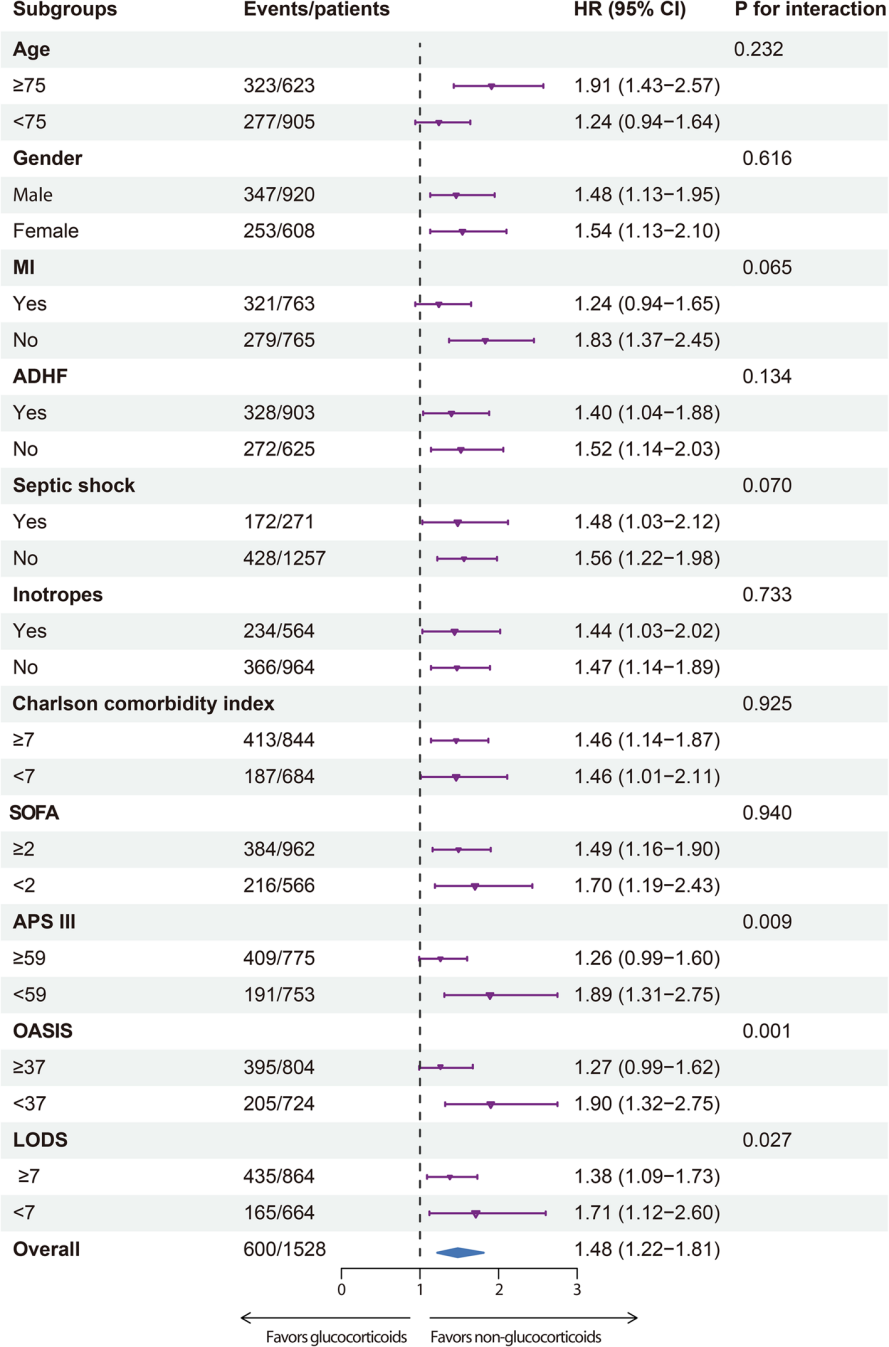
Subgroup analyses for the effects of glucocorticoid use on 90-day all-cause mortality. MI, myocardial infarction; ADHF, acute compensated heart failure; HR, hazard ratio; CI, confidence interval

Figure 3 shows subgroup analysis based on age, gender, MI, ADHF, septic shock, inotrope therapy, and ICU scoring systems in the pre-matched cohort. Multivariable Cox regression showed that the association between glucocorticoid use and 90-day all-cause mortality was consistent irrespective of age, gender, the presence of myocardial infarction, acute decompensated heart failure and septic shock, and inotrope therapy (all *P* for interaction > 0.05), but was more evident in low-risk patients as assessed by most ICU scoring systems, including APS III, OASIS, and LODS (all P for interaction ≤ 0.027). Supplementary Table S6 reveals sensitivity analyses for 90-day all-cause mortality regarding daily dosage, total dosage and exposure duration of glucocorticoids. Multivariable Cox regression analyses showed that the detrimental effect of glucocorticoids in CS patients was rarely dependent on these pharmacological properties. After excluding patients with chronic pulmonary disease and rheumatic disease, there was also an increase in 90-day all-cause mortality risk in glucocorticoid users compared with non-glucocorticoid users (HR 1.35, 95% CI 1.03–1.76; *P* = 0.028). We used raw data to further assess the robustness of our results. Similarly, glucocorticoid use (HR 1.47, 95% CI 1.19–1.83; *P* < 0.001) was significantly associated with an increased risk of 90-day all-cause mortality.

## Discussion

To the best of our knowledge, this is the largest study to investigate the prescription rate of short-term systemic use of glucocorticoids during hospitalization in CS patients and adverse events related to glucocorticoid use so far. Our study had several key findings. One in six patients with CS were exposed to glucocorticoids during admission. The decision to these agents was affected by several factors, such as age, heart rate, presence of rheumatic disease, chronic pulmonary disease and septic shock, and medical interventions with inotropes, CRRT, and mechanical ventilation. Unfortunately, glucocorticoid use did not result in an improved outcome and was associated with an increased risk of 90-day all-cause mortality. The increased risk of mortality was consistent regardless of age, gender, cause (i.e., MI and ADHF), the presence of septic shock, and inotrope therapy, but was more evident in low-risk patients as assessed by most ICU scoring systems, including APS III, OASIS, and LODS. Moreover, our finding did not vary across the pharmacological properties of glucocorticoids, including daily dosage, cumulative dosages, and exposure duration. In addition, glucocorticoid use was associated with an increased risk of hyperglycemia, but not infection.

Although the 2016 ESC guideline has stated that systemic use of glucocorticoids should be cautiously administrated in heart failure, given the assumption that causes water and sodium retention and leads a worsening condition [21], clinical evidence is lacking to support this thought. In real-world data, our study revealed that approximately 16% of patients with CS received short-term oral or intravenous glucocorticoid agents during hospitalization, and the determination of these drugs for clinical physicians was affected by many factors. Some comorbidities, as we observed, such as chronic pulmonary disease and rheumatic disease, were more prevalent in glucocorticoid users. Indeed, systemic administration of glucocorticoids is recommended to be an alternative for treating these comorbidities [10]. Additionally, we also observed that glucocorticoid therapy combined with CRRT and mechanical ventilation was a frequent strategy for CS patients. This was not surprising because, for example, in septic shock, the understanding of glucocorticoid administration for physicians depends on the severity of the disease [29]. Currently, limited studies reported epidemiological data on the systemic use of glucocorticoids in CS patients during hospitalization. Data from a registry study in Emergency Department showed that the prescription rate of the systemic use of glucocorticoids was 14% in patients with acute heart failure [22], which is consistent with what our study observed. Of concern, our study displayed that glucocorticoid users were younger but suffered from more premature deaths than non-glucocorticoid users, highlighting the need for a deeper investigation of the effects of glucocorticoid exposure on the prognosis of CS patients.

In the study, we found that systemic use of glucocorticoids was markedly associated with an increased risk of 90-day all-cause mortality. Nevertheless, there were many unbalanced variables regarding baseline characteristics, especially more frequent instrument interventions in glucocorticoid uses. A key question needed to be addressed whether glucocorticoid exposure was an indicator of the disease severity for CS. Multivariable regression and PSM analyses were used to adjust confounding factors, and both the adjustments showed that glucocorticoid use was an independent risk factor for 90-day all-cause mortality in CS. Furthermore, multivariable Logistic regression showed that most ICU scoring systems, including SOFA, OASIS, APS III, and LODS, did not support the hypothesis that glucocorticoids were prescribed to high-risk CS patients (Table 2). Therefore, glucocorticoid prescriptions could not merely reflect the disease severity of CS, and they should be used with caution in CS patients regardless of the disease severity.

For CS patients, vasoactive agents and inotropes, such as norepinephrine, dobutamine and milrinone, are widely used to maintain hemodynamic stability. To achieve the therapeutic goal of the maintaining hemodynamics, these agents inevitably induce sympathetic activation, resulting in tachycardia, elevated cardiac oxygen consumption, and increased risk of malignant arrhythmias [30, 31]. On the contrary, glucocorticoids have been shown to improve cardiac function, raise blood pressure and elevate diuretic response without the cost of increasing heart rate [32, 33]. However, the positive pharmacological effects of glucocorticoids did not translate into a survival benefit in our study. Similarly, in septic shock, although glucocorticoids are recommended to treat patients with the severe forms when stable hemodynamics cannot be maintained by vasopressor therapy and fluid resuscitation because they reduce the need of vasopressors and shorten the time to resolution of shock [29, 34], the recent meta-analysis has shown that low-dose glucocorticoid therapy was not associated with a decreased risk of mortality [35].

In our study, the increased risk of mortality related to glucocorticoid therapy may be interpreted in terms of its associated adverse effects. The detrimental effects of the short-term use of glucocorticoids have been documented in previous studies [14, 15, 34]. Briefly, glucocorticoids can induce changes of the immune system, leading to immunosuppression, which may potentially increase the risk of infection [15]. Our study observed more positive bacterial culture events in glucocorticoid users. After adjusting confounders, glucocorticoid prescription was not an independent risk factor of infection. This finding was consistent with the results in septic shock, where low-dose glucocorticoids were not associated with an increased risk of secondary infection [34]. In spite of these results, glucocorticoids might still increase the risk of infection in CS when clinicians do not promptly manage these confounding factors (e.g., instrumental treatment). Metabolism disturbance is another side effect of glucocorticoids, including hyperglycemia. To this point, acute hyperglycemic disorders are cardiotoxic, leading to numerous harmful effects. It is reported that hyperglycemia is responsible for cardiomyocyte and endothelial dysfunction, thus affecting cardiac function [6]. Acute hyperglycemia also creates a procoagulant effect by altering the activity of circulating tissue factors and procoagulation proteins [36, 37]. Moreover, acute hyperglycemia induces the production of oxidative stress, which leads to cardiomyocyte apoptosis and a decline in myocardial contractility [38]. Indeed, observational studies have reported that both admission and peak serum glucose level were independent predictors of mortality in CS irrespective of the presence of diabetes [6, 39]. In our study, hyperglycemia caused by glucocorticoids may somewhat interpret the increased mortality risk in glucocorticoid users. On the other hand, when glucocorticoid exposure in CS is inevitable in some specific conditions, interventions against these side effects might be especially important. It was recommended that blood glucose levels should be controlled between 144 and 180 mg/dL for CS patients [40].

Few studies have investigated the association of the systemic use of glucocorticoids with CS patients. A previous study reported that glucocorticoid administration had a detrimental effect on the prognosis of CS [9]. However, this study only included 35 patients and was not powered to determine the effect of glucocorticoids on the outcomes. With respect to heart failure, the SEMI-COVID-19 trial [41] enrolled 1155 heart failure patients hospitalized for COVID-19, most of whom were treated with intravenous methylprednisolone or dexamethasone. That study demonstrated that glucocorticoid use did not only result in heart failure deterioration, but also was associated with increased risks of in-hospital mortality, as well as mechanical ventilation and in-hospital complications, which was consistent with our finding. Nevertheless, another registry study (CORTicosterioids in Acute Heart Failure [CORT-AHF]) including 11,356 patients showed that glucocorticoid use in Emergency Department was not associated with changes for 90-day all-cause mortality in acute heart failure [22]. These inconsistent results might refer to the difference in mortality rate among the studies. In the control groups, the in-hospital and 90-day all-cause mortality rates in the CORT-AHF study were 7.0% and 16.3%, respectively, which was much lower than in our study (90-day mortality: 39.3%) and lower than in the SEMI-COVID-19 study (in-hospital mortality: 42.2%).

To date, a randomized controlled trial is in progress to investigate the association between low-dose glucocorticoid therapy and short-term endpoints for CS patients [42]. Actually, the focus of our study is somewhat distinct from this protocol. Our study was from real-world data and the primary outcome in our study was 90-day mortality, while the primary endpoint of the protocol was time to shock reversal. In addition, this protocol only enrolls the classic type of CS as defined by the Society for Cardiovascular Angiography and Intervention (SCAI) [43], and we also included patients at an earlier stage. Meanwhile, our study provides valuable evidence that interventions against glucocorticoid-related side effects may be the key to the success of the trial. Even so, we look forward to an early publication of the study.

There are several limitations to our study. Firstly, observational studies have a bias by nature, and our results might be affected by unmeasured confounding factors though they have been adjusted by multivariable regression analyses and PSM. Secondly, because of the inherent drawbacks in the MIMIC database, our study did not include inflammatory markers, nor did we investigate other side effects of glucocorticoids, such as thrombosis and gastrointestinal bleeding. Thirdly, full details of what led to death in CS are lacking, which might conceal the eventual association of glucocorticoid use with cardiovascular death. Fourthly, we identified CS subjects using ICD codes, and the heterogeneity in diagnostic criteria might not be ruled out. A classified diagnostic scheme for CS proposed by SCAI addresses the question about the diagnostic criteria [43]. Our study cannot accurately assess the disease severity for CS based on the SCAI classification because of limited data on physical examination and hemodynamics. Despite this, we used other ICU scoring systems as an alternative and we found that system use of glucocorticoids was more harmful in low-risk CS patients. Fifthly, the prevailing causes of CS were MI and ADHF, and our results might not be extrapolated into other uncommon causes. Finally, our results are derived from a single center and multicenter studies are required.

In conclusion, short-term systemic use of glucocorticoids was common in CS patients. These prescriptions were frequently administrated to CS patients with rapid heart rate, the presence of rheumatic disease, chronic pulmonary disease and septic shock, and the requirements of mechanical ventilation or continuous renal replacement therapy. However, glucocorticoid therapy was associated with increased risks of adverse events.

## Supplementary Information

**Additional file 1:** **Table S1.** International Classification of Diseases (ICD) of 9^th^ or 10^th^ codes for identifying specific diagnoses. **Table S2.** Multicollinearity analysis using linear regression model. **Table S3.** Logistic regression analysis for hyperglycemia in cardiogenic shock patients before matching. **Table S4.** Logistic regression analysis for hyperglycemia in cardiogenic shock patients after matching. **Table S5.** Logistic regression analysis for infection in cardiogenic shock patients before matching. **Table S6.** Sensitivity analysis for association of glucocorticoid exposure with 90-day all-cause mortality. **Figure S1.** Standardized mean difference of variables in pre-matched and post-matched cohorts.

## Data Availability

All data generated or analyzed during this study are available from the corresponding author upon reasonable request.

## Abbreviations

CS: Cardiogenic shock
MIMIC-IV: Medical information mart for intensive care IV
PSM: Propensity score matching
MI: Myocardial infarction
ADHF: Acute compensated heart failure
ICD: International classification of diseases
ICU: Intensive care unit
CRRT: Continuous renal replacement therapy
MCS: Mechanical circulatory support
SOFA: Sequential organ failure assessment
OASIS: Oxford acute severity of illness score
APS III: Acute physiology score III
LODS: Logistic organ dysfunction system
SD: Standard deviation
IQR: Interquartile range
SMD: Standardized mean difference
VIF: Variance inflation factor
HRs: Hazard ratios
ORs: Odds ratios
CIs: Confidence intervals
SCAI: The society for cardiovascular angiography and intervention

## References

[1] Bruno RR, Wolff G, Kelm M, Jung C (2022). Pharmacological treatment of cardiogenic shock - a state of the art review. Pharmacol Ther.

[2] Sinha SS, Rosner CM, Tehrani BN, Maini A, Truesdell AG, Lee SB, Bagchi P, Cameron J, Damluji AA, Desai M (2022). Cardiogenic shock from heart failure versus acute myocardial infarction: clinical characteristics, hospital course, and 1-year outcomes. Circ Heart Fail.

[3] Krychtiuk KA, Vrints C, Wojta J, Huber K, Speidl WS (2022). Basic mechanisms in cardiogenic shock: part 1-definition and pathophysiology. Eur Heart J Acute Cardiovasc Care.

[4] Jentzer JC, van Diepen S, Barsness GW, Henry TD, Menon V, Rihal CS, Naidu SS, Baran DA (2019). Cardiogenic shock classification to predict mortality in the cardiac intensive care unit. J Am Coll Cardiol.

[5] Chioncel O, Mebazaa A, Harjola VP, Coats AJ, Piepoli MF, Crespo-Leiro MG, Laroche C, Seferovic PM, Anker SD, Ferrari R (2017). Clinical phenotypes and outcome of patients hospitalized for acute heart failure: the ESC Heart Failure Long-Term Registry. Eur J Heart Fail.

[6] Scheen M, Giraud R, Bendjelid K (2021). Stress hyperglycemia, cardiac glucotoxicity, and critically ill patient outcomes current clinical and pathophysiological evidence. Physiol Rep.

[7] Bagate F, Lellouche N, Lim P, Moutereau S, Razazi K, Carteaux G, de Prost N, Dubois-Randé JL, Brun-Buisson C, Mekontso DA (2017). Prognostic value of relative adrenal insufficiency during cardiogenic shock: a prospective cohort study with long-term follow-up. Shock.

[8] Ducrocq N, Biferi P, Girerd N, Latar I, Lemoine S, Perez P, Thivilier C, Levy B, Kimmoun A (2018). Critical illness-related corticosteroid insufficiency in cardiogenic shock patients: prevalence and prognostic role. Shock.

[9] Tol MM, Shekar K, Barnett AG, McGree J, McWhinney BC, Ziegenfuss M, Ungerer JP, Fraser JF (2014). A preliminary investigation into adrenal responsiveness and outcomes in patients with cardiogenic shock after acute myocardial infarction. J Crit Care.

[10] Nussinovitch U, de Carvalho JF, Pereira RM, Shoenfeld Y (2010). Glucocorticoids and the cardiovascular system: state of the art. Curr Pharm Des.

[11] MacLeod C, Hadoke PWF, Nixon M (2021). Glucocorticoids: fuelling the fire of atherosclerosis or therapeutic extinguishers?. Int J Mol Sci.

[12] Liu B, Zhang TN, Knight JK, Goodwin JE (2019). The Glucocorticoid receptor in cardiovascular health and disease. Cells.

[13] Annane D, Pastores SM, Rochwerg B, Arlt W, Balk RA, Beishuizen A, Briegel J, Carcillo J, Christ-Crain M, Cooper MS (2017). Guidelines for the diagnosis and management of critical illness-related corticosteroid insufficiency (CIRCI) in critically ill patients (Part I): Society of Critical Care Medicine (SCCM) and European Society of Intensive Care Medicine (ESICM) 2017. Intensive Care Med.

[14] Yao TC, Huang YW, Chang SM, Tsai SY, Wu AC, Tsai HJ. Association between oral corticosteroid bursts and severe adverse events : a nationwide population-based cohort study. Ann Intern Med. 2020;173:325–30. 10.7326/m20-0432. 10.7326/M20-0432 32628532

[15] Waljee AK, Rogers MA, Lin P, Singal AG, Stein JD, Marks RM, Ayanian JZ, Nallamothu BK (2017). Short term use of oral corticosteroids and related harms among adults in the United States: population based cohort study. Bmj.

[16] Liu C, Liu G, Zhou C, Ji Z, Zhen Y, Liu K (2007). Potent diuretic effects of prednisone in heart failure patients with refractory diuretic resistance. Can J Cardiol.

[17] Liu C, Liu K (2014). Cardiac outcome prevention effectiveness of glucocorticoids in acute decompensated heart failure: COPE-ADHF study. J Cardiovasc Pharmacol.

[18] Aslam R, Ducrocq N, Thivilier C, Perez P, Gerard A, Kimmoun A, Levy B (2013). Critical illness-related corticosteroid insufficiency in cardiogenic shock. Br J Anaesth.

[19] Wolff B, Machill K, Schulzki I, Schumacher D, Werner D (2007). Acute reversible cardiomyopathy with cardiogenic shock in a patient with Addisonian crisis: a case report. Int J Cardiol.

[20] Mekontso-Dessap A, Marrache D, Vieillard-Baron A (2005). Images in cardiology: acute adrenal insufficiency complicated by cardiogenic shock. Heart.

[21] Ponikowski P, Voors AA, Anker SD, Bueno H, Cleland JGF, Coats AJS, Falk V, González-Juanatey JR, Harjola VP, Jankowska EA (2016). 2016 ESC Guidelines for the diagnosis and treatment of acute and chronic heart failure: the task force for the diagnosis and treatment of acute and chronic heart failure of the European Society of Cardiology (ESC)developed with the special contribution of the Heart Failure Association (HFA) of the ESC. Eur Heart J.

[22] Miró Ò, Takagi K, Gayat E, Llorens P, Martín-Sánchez FJ, Jacob J, Herrero-Puente P, Gil V, Wussler DN, Richard F (2019). CORT-AHF study: effect on outcomes of systemic corticosteroid therapy during early management acute heart failure. JACC Heart Fail.

[23] von Elm E, Altman DG, Egger M, Pocock SJ, Gøtzsche PC, Vandenbroucke JP (2014). The Strengthening the Reporting of Observational Studies in Epidemiology (STROBE) Statement: guidelines for reporting observational studies. Int J Surg.

[24] Prete A, Bancos I (2021). Glucocorticoid induced adrenal insufficiency. Bmj.

[25] Abdin A, Pöss J, Fuernau G, Ouarrak T, Desch S, Eitel I, de Waha S, Zeymer U, Böhm M, Thiele H (2018). Correction to: prognostic impact of baseline glucose levels in acute myocardial infarction complicated by cardiogenic shock-a substudy of the IABP-SHOCK II-trial. Clin Res Cardiol.

[26] Shah AD, Bartlett JW, Carpenter J, Nicholas O, Hemingway H (2014). Comparison of random forest and parametric imputation models for imputing missing data using MICE: a CALIBER study. Am J Epidemiol.

[27] Huang YH, Cai WK, Yin SJ, Wang P, Li ZR, Yang Q, Zhou T, Meng R, Yang M, Guo Y (2022). Histamine H2 receptor antagonist exposure was related to decreased all-cause mortality in critical ill patients with heart failure: a cohort study. Eur J Prev Cardiol.

[28] González Ariza A, ArandoArbulu A, León Jurado JM, Navas González FJ, Delgado Bermejo JV, Camacho Vallejo ME (2021). Discriminant canonical tool for differential biometric characterization of multivariety endangered hen breeds. Animals.

[29] Vandewalle J, Libert C (2020). Glucocorticoids in sepsis: to be or not to be. Front Immunol.

[30] Metra M, Chioncel O, Cotter G, Davison B, Filippatos G, Mebazaa A, Novosadova M, Ponikowski P, Simmons P, Soffer J (2022). Safety and efficacy of istaroxime in patients with acute heart failure-related pre-cardiogenic shock - a multicentre, randomized, double-blind, placebo-controlled, parallel group study (SEISMiC). Eur J Heart Fail.

[31] McDonagh TA, Metra M, Adamo M, Gardner RS, Baumbach A, Böhm M, Burri H, Butler J, Čelutkienė J, Chioncel O (2021). 2021 ESC Guidelines for the diagnosis and treatment of acute and chronic heart failure. Eur Heart J.

[32] Bagate F, Coppens A, Masi P, de Prost N, Carteaux G, Razazi K, Mekontso DessapA (2022). Cardiac and vascular effects of low-dose steroids during the early phase of septic shock: an echocardiographic study. Front Cardiovasc Med.

[33] Li S, Zhao Q, Zhen Y, Li L, Mi Y, Li T, Liu K, Liu C (2021). The impact of glucocorticoid therapy on guideline-directed medical treatment titration in patients hospitalized for heart failure with low blood pressure: a retrospective study. Int J Gen Med.

[34] Chan ED, Chan MM, Chan MM, Marik PE (2020). Use of glucocorticoids in the critical care setting: Science and clinical evidence. Pharmacol Ther.

[35] Fujii T, Salanti G, Belletti A, Bellomo R, Carr A, Furukawa TA, Luethi N, Luo Y, Putzu A, Sartini C (2022). Effect of adjunctive vitamin C, glucocorticoids, and vitamin B1 on longer-term mortality in adults with sepsis or septic shock: a systematic review and a component network meta-analysis. Intensive Care Med.

[36] Vaidyula VR, Rao AK, Mozzoli M, Homko C, Cheung P, Boden G (2006). Effects of hyperglycemia and hyperinsulinemia on circulating tissue factor procoagulant activity and platelet CD40 ligand. Diabetes.

[37] Lemkes BA, Hermanides J, Devries JH, Holleman F, Meijers JC, Hoekstra JB (2010). Hyperglycemia: a prothrombotic factor?. J Thromb Haemost.

[38] Dambrova M, Zuurbier CJ, Borutaite V, Liepinsh E, Makrecka-Kuka M (2021). Energy substrate metabolism and mitochondrial oxidative stress in cardiac ischemia/reperfusion injury. Free Radic Biol Med.

[39] Kataja A, Tarvasmäki T, Lassus J, Cardoso J, Mebazaa A, Køber L, Sionis A, Spinar J, Carubelli V, Banaszewski M (2017). The association of admission blood glucose level with the clinical picture and prognosis in cardiogenic shock - results from the cardshock study. Int J Cardiol.

[40] Thiele H, Ohman EM, de Waha-Thiele S, Zeymer U, Desch S (2019). Management of cardiogenic shock complicating myocardial infarction: an update 2019. Eur Heart J.

[41] Pérez-Belmonte LM, Sanz-Cánovas J, Salinas A, Fornie IS, Méndez-Bailón M, Gómez-Huelgas R, the S-C-N (2021). Corticosteroid therapy in patients with heart failure hospitalized for COVID-19: a multicenter retrospective study. Int Emerg Med.

[42] MekontsoDessap A, Bagate F, Delmas C, Morichau-Beauchant T, Cholley B, Cariou A, Lattuca B, Moussa M, Mongardon N, Fard D (2022). Low-dose corticosteroid therapy for cardiogenic shock in adults (COCCA): study protocol for a randomized controlled trial. Trials.

[43] Baran DA, Grines CL, Bailey S, Burkhoff D, Hall SA, Henry TD, Hollenberg SM, Kapur NK, O’Neill W, Ornato JP, et al. SCAI clinical expert consensus statement on the classification of cardiogenic shock: This document was endorsed by the American College of Cardiology (ACC), the American Heart Association (AHA), the Society of Critical Care Medicine (SCCM), and the Society of Thoracic Surgeons (STS) in April 2019. Catheter Cardiovasc Interv. 2019;94:29–37. 10.1002/ccd.28329. 10.1002/ccd.28329 31104355

