# Supplementary material for: Association between short-term systemic use of glucocorticoids and prognosis of cardiogenic shock: a retrospective analysis
Source: BMC Anesthesiol. 2023 May 18;23:169. doi: 10.1186/s12871-023-02131-y (PMC10193317; doi:10.1186/s12871-023-02131-y)
Supplement: Supplementary file 1 — Additional file 1: Table S1. International Classification of Diseases (ICD) of 9th or 10th codes for identifying specific diagnoses. Table S2. Multicollinearity analysis using linear regression model. Table S3. Logistic regression analysis for hyperglycemia in cardiogenic shock patients before matching. Table S4. Logistic regression analysis for hyperglycemia in cardiogenic shock patients after matching. Table S5. Logistic regression analysis for infection in cardiogenic shock patients before matching. Table S6. Sensitivity analysis for association of glucocorticoid exposure with 90-day all-cause mortality. Figure S1. Standardized mean difference of variables in pre-matched and post-matched cohorts. [file 12871_2023_2131_MOESM1_ESM.docx]

**Supplementary data**

**Table S1.** International Classification of Diseases (ICD) of 9^th^ or 10^th^ codes for identifying specific diagnoses.

**Table S2.** Multicollinearity analysis using linear regression model.

**Table S3.** Logistic regression analysis for hyperglycemia in cardiogenic shock patients before matching.

**Table S4.** Logistic regression analysis for hyperglycemia in cardiogenic shock patients after matching.

**Table S5.** Logistic regression analysis for infection in cardiogenic shock patients before matching.

**Table S6.** Sensitivity analysis for association of glucocorticoid exposure with 90-day all-cause mortality.

**Figure S1.** Standardized mean difference of variables in pre-matched and post-matched cohorts.

**Table S1.** International Classification of Diseases (ICD) of 9^th^ or 10^th^ codes for identifying specific diagnoses.

| **Diagnoses** | **ICD codes** |
| --- | --- |
| **Cardiogenic shock** | 78551, 99801, R570, T8111, T8111XA, T8111XD |
| **Steroid use history** | V5865, V8745, Z795, Z7952, Z92241, Z9224 |

**Table S2.** **Multicollinearity analysis using linear regression model.**

| Variables | VIFs | |
| --- | --- | --- |
|  | Before removal | After removal |
| Age | 2.09 | 2.09 |
| Male | 1.30 | 1.27 |
| BMI | 1.15 | 1.14 |
| Charlson comorbidity index | 3.18 | 3.17 |
| SOFA | 1.31 | 1.31 |
| OASIS | 3.32 | 3.30 |
| APS Ⅲ | 4.30 | 4.28 |
| LODS | 4.49 | 4.47 |
| SBP | 1.64 | 1.64 |
| DBP | 1.82 | 1.81 |
| Heart rate | 1.27 | 1.27 |
| Hypertension | 1.44 | 1.44 |
| Diabetes | 1.62 | 1.60 |
| Dyslipidemia | 1.24 | 1.24 |
| CKD | 2.46 | 2.45 |
| Rheumatic disease | 1.06 | 1.06 |
| Chronic pulmonary disease | 1.18 | 1.17 |
| ADHF | 1.23 | 1.23 |
| MI | 1.60 | 1.59 |
| Cardiac arrest | 1.16 | 1.15 |
| Septic shock | 1.26 | 1.26 |
| Lactate | 1.57 | 1.56 |
| WBC | 1.33 | 1.32 |
| Platelet | 1.25 | 1.23 |
| RBC | 5.25 | 1.40 |
| **Hemoglobin** | 5.42 | **Removed** |
| AST | 3.93 | 3.93 |
| ALT | 3.98 | 3.98 |
| Albumin | 1.33 | 1.31 |
| BUN | 2.42 | 2.40 |
| Creatinine | 2.28 | 2.28 |
| Bicarbonate | 2.60 | 2.34 |
| pH | 11.25 | 1.13 |
| **BE** | 11.59 | **Removed** |
| Glucose | 1.52 | 1.50 |
| Sodium | 3.47 | 3.44 |
| Potassium | 1.40 | 1.38 |
| Chloride | 4.50 | 4.46 |
| Calcium | 1.38 | 1.37 |
| Magnesium | 1.21 | 1.21 |
| INR | 1.19 | 1.19 |
| PTT | 1.20 | 1.20 |
| Antiplatelet | 1.51 | 1.51 |
| Statins | 1.69 | 1.69 |
| Inotropes | 1.27 | 1.27 |
| Vasopressors | 1.59 | 1.59 |
| MCS | 1.20 | 1.20 |
| CRRT | 1.40 | 1.40 |
| Revascularization | 1.47 | 1.47 |
| Mechanical ventilation | 2.28 | 2.27 |
| Glucocorticoids | 1.16 | 1.15 |
| **Mean** | 2.43 | 1.88 |

VIFs, variance inflation factors; BMI, body mass index; SOFA, sequential organ failure assessment; OASIS, oxford acute severity of illness score; APS Ⅲ, acute physiology score Ⅲ; LODS, logistic organ dysfunction system; SBP, systolic blood pressure; DBP, diastolic blood pressure; CKD, chronic kidney disease; ADHF, acute decompensated heart failure; MI, myocardial infarction; WBC, white blood cell; RBC, red blood cell; AST, aspartate aminotransferase; ALT, alanine aminotransferase; BUN, blood urea nitrogen; BE, base excess; INR, international normalized ratio; PTT, partial thromboplastin time; MCS, mechanical circulatory support; CRRT, continuous renal replacement therapy.

**Table S3.** Logistic regression analysis for hyperglycemia in cardiogenic shock patients before matching.

|  | **Univariable regression** | | **Multivariable regression** | |
| --- | --- | --- | --- | --- |
|  | **OR (95% CI)** | **P value** | **OR (95% CI)** | **P value** |
| Age, per 1 year | 1.00 (0.99–1.01) | 0.797 | – |  |
| Male, yes | 1.02 (0.83–1.25) | 0.857 | – |  |
| BMI, per 1 Kg/m^2^ | 1.01 (0.99–1.02) | 0.267 | – |  |
| Charlson comorbidity index,  per 1 score | 1.11 (1.07–1.16) | <0.001 | 1.00 (0.94–1.07) | 0.972 |
| SOFA, per 1 score | 1.04 (1.00–1.08) | 0.046 | 0.97 (0.92–1.02) | 0.209 |
| OASIS, per 1 score | 1.04 (1.03–1.05) | <0.001 | 0.98 (0.96–1.01) | 0.178 |
| **APS Ⅲ, per 1 score** | 1.02 (1.02–1.03) | <0.001 | 1.02 (1.01–1.03) | 0.001 |
| LODS, per 1 score | 1.15 (1.12–1.18) | <0.001 | 1.01 (0.94–1.08) | 0.836 |
| SBP, per 1 mmHg | 1.00 (1.00–1.01) | 0.622 | – |  |
| DBP, per 1 mmHg | 1.00 (1.00–1.01) | 0.305 | – |  |
| Heart rate, per 1 bpm | 1.01 (1.00–1.01) | 0.003 | 1.00 (1.00–1.01) | 0.527 |
| Hypertension | 0.90 (0.72–1.12) | 0.355 | – |  |
| **Diabetes** | 4.24 (3.38–5.33) | <0.001 | 2.83 (2.08–3.85) | <0.001 |
| Dyslipidemia | 1.25 (1.02–1.53) | 0.028 | 1.03 (0.79–1.34) | 0.854 |
| CKD | 1.36 (1.1–1.68) | 0.004 | 0.92 (0.63–1.32) | 0.639 |
| Rheumatic disease | 0.57 (0.31–1.05) | 0.070 | – |  |
| Chronic pulmonary disease | 0.95 (0.76–1.19) | 0.658 | – |  |
| **ADHF** | 1.31 (1.07–1.60) | 0.010 | 1.61 (1.22–2.13) | 0.001 |
| MI | 1.22 (1.00–1.49) | 0.051 | – |  |
| **Cardiac arrest** | 3.03 (2.16–4.27) | <0.001 | 2.69 (1.76–4.13) | <0.001 |
| Septic shock | 1.80 (1.37–2.37) | <0.001 | 1.07 (0.75–1.55) | 0.699 |
| Lactate, per 1 mmol/L | 1.23 (1.16–1.29) | <0.001 | 1.07 (1.00–1.15) | 0.059 |
| WBC, per 1×10^9^/L | 1.03 (1.01–1.04) | 0.001 | 1.00 (0.98–1.02) | 0.892 |
| Platelet, per 1×10^9^/L | 1.00 (1.00–1.00) | 0.465 | – |  |
| RBC, per 1×10^12^/L | 1.09 (0.96–1.23) | 0.185 | – |  |
| Hemoglobin, per 1 g/L | 1.01 (0.97–1.05) | 0.656 | – |  |
| AST, per 1 IU/L | 1.00 (1.00–1.00) | 0.006 | 1.00 (1.00–1.00) | 0.986 |
| ALT, per 1 IU/L | 1.00 (1.00–1.00) | 0.020 | 1.00 (1.00–1.00) | 0.958 |
| Albumin, per 1 g/dL | 0.79 (0.66–0.93) | 0.005 | 0.98 (0.78–1.23) | 0.868 |
| BUN, per 1 mg/dL | 1.01 (1.01–1.01) | <0.001 | 1.01 (1.00–1.02) | 0.008 |
| Creatinine, per 1 mg/dL | 1.09 (1.01–1.17) | 0.020 | 0.88 (0.78–1.00) | 0.055 |
| Bicarbonate, per 1 mmol/L | 0.95 (0.93–0.97) | <0.001 | 1.00 (0.96–1.04) | 0.991 |
| pH, per 0.01 | 0.25 (0.1–0.64) | 0.004 | 1.38 (0.48–4.02) | 0.550 |
| BE, per 1 mmol/L | 0.98 (0.96–1.00) | 0.031 | – |  |
| **Glucose, per 1 mg/dL** | 1.02 (1.01–1.02) | <0.001 | 1.02 (1.01–1.02) | <0.001 |
| Sodium, per 1 mmol/L | 0.98 (0.96–1.00) | 0.014 | 1.01 (0.97–1.06) | 0.560 |
| Potassium, per 1 mmol/L | 1.14 (1.02–1.29) | 0.025 | 0.99 (0.83–1.17) | 0.877 |
| Chloride, per 1 mmol/L | 0.98 (0.96–0.99) | 0.002 | 0.99 (0.96–1.03) | 0.717 |
| Calcium, per 1 mg/dL | 0.85 (0.76–0.95) | 0.005 | 0.94 (0.80–1.11) | 0.460 |
| Magnesium, per 1 mg/dL | 0.95 (0.77–1.18) | 0.649 | – |  |
| INR, per 0.1 | 1.03 (0.95–1.12) | 0.425 | – |  |
| PTT, per 1 s | 1.01 (1.00–1.01) | 0.001 | 1.00 (1.00–1.01) | 0.518 |
| Antiplatelet | 1.07 (0.83–1.38) | 0.586 | – |  |
| Statins | 1.04 (0.84–1.30) | 0.705 | – |  |
| Inotropes | 1.43 (1.16–1.76) | 0.001 | 1.24 (0.93–1.65) | 0.141 |
| Vasopressors | 1.70 (1.34–2.16) | <0.001 | 1.02 (0.72–1.46) | 0.895 |
| MCS | 1.63 (1.11–2.39) | 0.013 | 0.98 (0.58–1.64) | 0.934 |
| **CRRT** | 3.04 (2.20–4.19) | <0.001 | 2.31 (1.49–3.59) | <0.001 |
| Revascularization | 1.01 (0.82–1.26) | 0.899 | – |  |
| Mechanical ventilation | 1.82 (1.48–2.25) | <0.001 | 1.12 (0.77–1.63) | 0.545 |
| **Glucocorticoids** | 2.33 (1.74–3.11) | <0.001 | 2.14 (1.48–3.10) | <0.001 |

BMI, body mass index; SOFA, sequential organ failure assessment; OASIS, oxford acute severity of illness score; APS Ⅲ, acute physiology score Ⅲ; LODS, logistic organ dysfunction system; SBP, systolic blood pressure; DBP, diastolic blood pressure; CKD, chronic kidney disease; ADHF, acute decompensated heart failure; MI, myocardial infarction; WBC, white blood cell; RBC, red blood cell; AST, aspartate aminotransferase; ALT, alanine aminotransferase; BUN, blood urea nitrogen; BE, base excess; INR, international normalized ratio; PTT, partial thromboplastin time; MCS, mechanical circulatory support; CRRT, continuous renal replacement therapy; OR, odds ratio; CI, confidence interval.

**Table S4.** Logistic regression analysis for hyperglycemia in cardiogenic shock patients after matching.

|  | **Univariable regression** | | **Multivariable regression** | |
| --- | --- | --- | --- | --- |
|  | **OR (95% CI)** | **P value** | **OR (95% CI)** | **P value** |
| Age, per 1 year | 1.00 (0.99–1.01) | 0.986 | – |  |
| Male, yes | 1.24 (0.91–1.70) | 0.177 | – |  |
| BMI, per 1 Kg/m^2^ | 1.02 (1.00–1.04) | 0.119 | – |  |
| Charlson comorbidity index,  per 1 score | 1.08 (1.02–1.14) | 0.009 | 1.00 (0.91–1.10) | 0.973 |
| SOFA, per 1 score | 1.05 (0.99–1.11) | 0.094 | – |  |
| OASIS, per 1 score | 1.04 (1.02–1.06) | <0.001 | 0.98 (0.95–1.02) | 0.379 |
| APS Ⅲ, per 1 score | 1.02 (1.01–1.03) | <0.001 | 1.01 (1.00–1.03) | 0.051 |
| LODS, per 1 score | 1.13 (1.08–1.18) | <0.001 | 0.99 (0.89–1.11) | 0.905 |
| SBP, per 1 mmHg | 1.00 (0.99–1.01) | 0.837 | – |  |
| DBP, per 1 mmHg | 1.01 (1.00–1.01) | 0.158 | – |  |
| Heart rate, per 1 bpm | 1.01 (1.00–1.01) | 0.040 | 1.01 (1.00–1.02) | 0.077 |
| Hypertension | 0.95 (0.67–1.34) | 0.753 | – |  |
| **Diabetes** | 5.24 (3.52–7.79) | <0.001 | 2.49 (1.44–4.33) | 0.001 |
| Dyslipidemia | 1.27 (0.93–1.74) | 0.137 | – |  |
| CKD | 1.51 (1.08–2.11) | 0.015 | 1.09 (0.63–1.89) | 0.748 |
| Rheumatic disease | 0.45 (0.21–0.94) | 0.035 | 0.60 (0.22–1.58) | 0.299 |
| Chronic pulmonary disease | 0.83 (0.60–1.14) | 0.254 | – |  |
| ADHF | 1.21 (0.88–1.65) | 0.237 | – |  |
| MI | 1.37 (1.00–1.87) | 0.051 | – |  |
| **Cardiac arrest** | 3.07 (1.88–5.01) | <0.001 | 2.70 (1.44–5.04) | 0.002 |
| Septic shock | 1.49 (1.03–2.14) | 0.033 | 1.41 (0.86–2.32) | 0.177 |
| Lactate, per 1 mmol/L | 1.21 (1.12–1.30) | <0.001 | 1.08 (0.98–1.19) | 0.115 |
| WBC, per 1×10^9^/L | 1.03 (1.01–1.06) | 0.013 | 0.98 (0.95–1.02) | 0.316 |
| Platelet, per 1×10^9^/L | 1.00 (1.00–1.00) | 0.643 | – |  |
| RBC, per 1×10^12^/L | 1.17 (0.96–1.44) | 0.121 | – |  |
| Hemoglobin, per 1 g/L | 1.06 (0.98–1.13) | 0.129 | – |  |
| AST, per 1 IU/L | 1.00 (1.00–1.00) | 0.314 | – |  |
| ALT, per 1 IU/L | 1.00 (1.00–1.00) | 0.291 | – |  |
| Albumin, per 1 g/dL | 1.01 (0.79–1.31) | 0.918 | – |  |
| BUN, per 1 mg/dL | 1.01 (1.00–1.02) | 0.001 | 1.01 (1.00–1.02) | 0.128 |
| Creatinine, per 1 mg/dL | 1.09 (0.97–1.22) | 0.135 | – |  |
| Bicarbonate, per 1 mmol/L | 0.94 (0.91–0.97) | <0.001 | 1.02 (0.96–1.08) | 0.494 |
| pH, per 0.01 | 0.76 (0.35–1.64) | 0.480 | – |  |
| BE, per 1 mmol/L | 1.00 (0.99–1.01) | 0.647 | – |  |
| **Glucose, per 1 mg/dL** | 1.02 (1.01–1.02) | <0.001 | 1.02 (1.01–1.02) | <0.001 |
| Sodium, per 1 mmol/L | 0.96 (0.93–0.99) | 0.013 | 0.95 (0.89–1.02) | 0.163 |
| Potassium, per 1 mmol/L | 1.13 (0.95–1.35) | 0.163 | – |  |
| Chloride, per 1 mmol/L | 0.97 (0.95–1.00) | 0.023 | 1.03 (0.97–1.09) | 0.326 |
| Calcium, per 1 mg/dL | 0.93 (0.79–1.09) | 0.365 | – |  |
| Magnesium, per 1 mg/dL | 0.97 (0.73–1.31) | 0.863 | – |  |
| INR, per 0.1 | 1.06 (0.89–1.25) | 0.536 | – |  |
| PTT, per 1 s | 1.00 (1.00–1.01) | 0.337 | – |  |
| Antiplatelet | 1.02 (0.70–1.49) | 0.918 | – |  |
| Statins | 1.16 (0.84–1.61) | 0.374 | – |  |
| Inotropes | 1.37 (0.98–1.92) | 0.066 | – |  |
| Vasopressors | 2.02 (1.34–3.04) | 0.001 | 1.03 (0.56–1.91) | 0.926 |
| MCS | 1.18 (0.73–1.92) | 0.491 | – |  |
| CRRT | 2.14 (1.41–3.24) | <0.001 | 1.67 (0.95–2.93) | 0.077 |
| Revascularization | 1.58 (1.09–2.30) | 0.016 | 1.62 (0.95–2.74) | 0.074 |
| Mechanical ventilation | 1.89 (1.32–2.71) | <0.001 | 1.50 (0.80–2.81) | 0.202 |
| **Glucocorticoids** | 1.86 (1.33–2.61) | <0.001 | 2.36 (1.54–3.62) | <0.001 |

BMI, body mass index; SOFA, sequential organ failure assessment; OASIS, oxford acute severity of illness score; APS Ⅲ, acute physiology score Ⅲ; LODS, logistic organ dysfunction system; SBP, systolic blood pressure; DBP, diastolic blood pressure; CKD, chronic kidney disease; ADHF, acute decompensated heart failure; MI, myocardial infarction; WBC, white blood cell; RBC, red blood cell; AST, aspartate aminotransferase; ALT, alanine aminotransferase; BUN, blood urea nitrogen; BE, base excess; INR, international normalized ratio; PTT, partial thromboplastin time; MCS, mechanical circulatory support; CRRT, continuous renal replacement therapy; OR, odds ratio; CI, confidence interval.

**Table S5.** Logistic regression analysis for infection in cardiogenic shock patients before matching.

|  | **Univariable regression** | | **Multivariable regression** | |
| --- | --- | --- | --- | --- |
|  | **OR (95% CI)** | **P value** | **OR (95% CI)** | **P value** |
| Age, per 1 year | 1.00 (0.99–1.01) | 0.502 | – |  |
| Male, yes | 0.80 (0.62–1.03) | 0.079 | – |  |
| BMI, per 1 Kg/m^2^ | 1.01 (0.99–1.03) | 0.166 | – |  |
| Charlson comorbidity index,  per 1 score | 1.02 (0.97–1.07) | 0.450 | – |  |
| SOFA, per 1 score | 1.10 (1.05–1.15) | <0.001 | 1.01 (0.96–1.06) | 0.678 |
| OASIS, per 1 score | 1.06 (1.04–1.07) | <0.001 | 1.00 (0.98–1.02) | 0.738 |
| APS Ⅲ, per 1 score | 1.02 (1.01–1.02) | <0.001 | 1.01 (1.00–1.01) | 0.238 |
| LODS, per 1 score | 1.18 (1.14–1.22) | <0.001 | 1.01 (0.94–1.09) | 0.724 |
| SBP, per 1 mmHg | 1.00 (0.99–1.00) | 0.549 | – |  |
| DBP, per 1 mmHg | 1.00 (0.99–1.01) | 0.909 | – |  |
| Heart rate, per 1 bpm | 1.00 (0.99–1.01) | 0.853 | – |  |
| Hypertension | 1.05 (0.80–1.38) | 0.714 | – |  |
| Diabetes | 0.91 (0.70–1.17) | 0.458 | – |  |
| Dyslipidemia | 0.74 (0.58–0.95) | 0.018 | 0.80 (0.61–1.04) | 0.098 |
| CKD | 1.08 (0.83–1.39) | 0.564 | – |  |
| Rheumatic disease | 0.79 (0.36–1.71) | 0.549 | – |  |
| Chronic pulmonary disease | 1.28 (0.98–1.66) | 0.066 | – |  |
| ADHF | 0.70 (0.55–0.90) | 0.005 | 0.80 (0.61–1.05) | 0.114 |
| MI | 0.96 (0.75–1.22) | 0.726 | – |  |
| Cardiac arrest | 1.13 (0.79–1.63) | 0.498 | – |  |
| **Septic shock** | 2.82 (2.12–3.75) | <0.001 | 1.83 (1.33–2.54) | <0.001 |
| Lactate, per 1 mmol/L | 0.99 (0.94–1.05) | 0.751 | – |  |
| WBC, per 1×10^9^/L | 1.02 (1.00–1.03) | 0.017 | 1.01 (0.99–1.02) | 0.496 |
| Platelet, per 1×10^9^/L | 1.00 (1.00–1.00) | 0.804 | – |  |
| RBC, per 1×10^12^/L | 0.91 (0.78–1.06) | 0.207 | – |  |
| Hemoglobin, per 1 g/L | 0.96 (0.92–1.02) | 0.170 | – |  |
| AST, per 1 IU/L | 1.00 (1.00–1.00) | 0.071 | – |  |
| ALT, per 1 IU/L | 1.00 (1.00–1.00) | 0.133 | – |  |
| Albumin, per 1 g/dL | 0.62 (0.50–0.76) | <0.001 | 0.84 (0.67–1.06) | 0.135 |
| BUN, per 1 mg/dL | 1.00 (1.00–1.01) | 0.483 | – |  |
| Creatinine, per 1 mg/dL | 1.06 (0.98–1.15) | 0.149 | – |  |
| Bicarbonate, per 1 mmol/L | 0.98 (0.96–1.01) | 0.230 | – |  |
| pH, per 0.01 | 0.91 (0.55–1.51) | 0.724 | – |  |
| BE, per 1 mmol/L | 1.00 (0.99–1.02) | 0.725 | – |  |
| Glucose, per 1 mg/dL | 1.00 (1.00–1.00) | 0.152 | – |  |
| Sodium, per 1 mmol/L | 1.00 (0.97–1.02) | 0.710 | – |  |
| Potassium, per 1 mmol/L | 1.10 (0.96–1.26) | 0.188 | – |  |
| Chloride, per 1 mmol/L | 0.99 (0.98–1.01) | 0.563 | – |  |
| Calcium, per 1 mg/dL | 0.85 (0.75–0.98) | 0.020 | 1.07 (0.93–1.24) | 0.347 |
| Magnesium, per 1 mg/dL | 1.00 (0.77–1.30) | 0.977 | – |  |
| INR, per 0.1 | 1.01 (0.92–1.11) | 0.810 | – |  |
| PTT, per 1 s | 1.00 (1.00–1.00) | 0.819 | – |  |
| Antiplatelet | 0.83 (0.61–1.12) | 0.229 | – |  |
| Statins | 0.95 (0.72–1.24) | 0.682 | – |  |
| Inotropes | 1.69 (1.32–2.17) | <0.001 | 1.31 (0.99–1.74) | 0.061 |
| Vasopressors | 4.08 (2.72–6.14) | <0.001 | 1.36 (0.84–2.20) | 0.206 |
| MCS | 1.93 (1.29–2.89) | 0.001 | 1.17 (0.75–1.82) | 0.498 |
| **CRRT** | 3.64 (2.68–4.93) | <0.001 | 1.83 (1.29–2.59) | 0.001 |
| Revascularization | 0.92 (0.71–1.21) | 0.565 | – |  |
| **Mechanical ventilation** | 4.70 (3.37–6.56) | <0.001 | 2.85 (1.87–4.34) | <0.001 |
| Glucocorticoids | 1.85 (1.37–2.51) | <0.001 | 1.23 (0.88–1.73) | 0.221 |

BMI, body mass index; SOFA, sequential organ failure assessment; OASIS, oxford acute severity of illness score; APS Ⅲ, acute physiology score Ⅲ; LODS, logistic organ dysfunction system; SBP, systolic blood pressure; DBP, diastolic blood pressure; CKD, chronic kidney disease; ADHF, acute decompensated heart failure; MI, myocardial infarction; WBC, white blood cell; RBC, red blood cell; AST, aspartate aminotransferase; ALT, alanine aminotransferase; BUN, blood urea nitrogen; BE, base excess; INR, international normalized ratio; PTT, partial thromboplastin time; MCS, mechanical circulatory support; CRRT, continuous renal replacement therapy; OR, odds ratio; CI, confidence interval.

**Table S6.** Sensitivity analysis for association of glucocorticoid exposure with 90-day all-cause mortality.

|  | **HR (95% CI)** | **P value** |
| --- | --- | --- |
| **Daily dosage** |  |  |
| Non-glucocorticoids | Reference |  |
| < 50mg/day | 1.48 (1.14–1.94) | 0.003 |
| ≥ 50mg/day | 1.42 (1.10–1.83) | 0.007 |
| **Cumulative dosages** |  |  |
| Non–glucocorticoids | Reference |  |
| < 180mg | 1.54 (1.19–1.97) | 0.001 |
| ≥ 180mg | 1.36 (1.04–1.78) | 0.023 |
| **Exposure duration** |  |  |
| Non-glucocorticoids | Reference |  |
| < 4 days | 1.71 (1.32–2.22) | <0.001 |
| ≥ 4 days | 1.25 (0.97–1.63) | 0.089 |

HR, hazard ratio; CI, confidence interval.

**Figure S1.** Standardized mean difference of variables in pre-matched and post-matched cohorts.

**
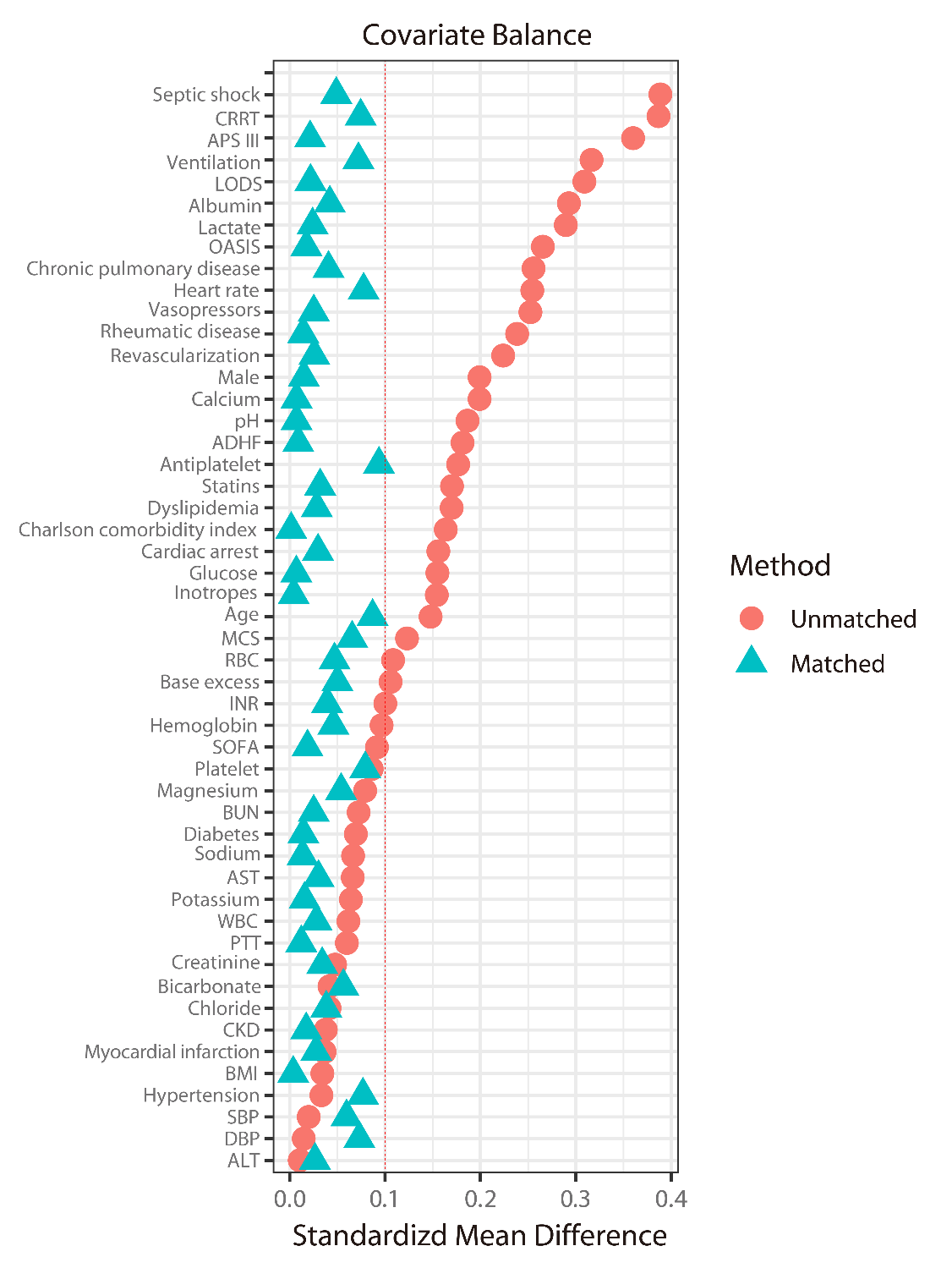
**

CRRT, continuous renal replacement therapy; APS Ⅲ, acute physiology score Ⅲ; LODS, logistic organ dysfunction system; OASIS, oxford acute severity of illness score; ADHF, acute decompensated heart failure; MCS, mechanical circulatory support; RBC, red blood cell; INR, international normalized ratio; SOFA, sequential organ failure assessment; BUN, blood urea nitrogen; AST, aspartate aminotransferase; WBC, white blood cell; PTT, partial thromboplastin time;

CKD, chronic kidney disease; BMI, body mass index; SBP, systolic blood pressure; DBP, diastolic blood pressure; ALT, alanine aminotransferase.
